# Attribution of global foodborne disease to specific foods: Findings from a World Health Organization structured expert elicitation

**DOI:** 10.1371/journal.pone.0183641

**Published:** 2017-09-14

**Authors:** Sandra Hoffmann, Brecht Devleesschauwer, Willy Aspinall, Roger Cooke, Tim Corrigan, Arie Havelaar, Frederick Angulo, Herman Gibb, Martyn Kirk, Robin Lake, Niko Speybroeck, Paul Torgerson, Tine Hald

**Affiliations:** 1 U.S. Dept. of Agriculture, Economic Research Service, Washington D.C., United States of America; 2 Department of Public Health and Surveillance, Scientific Institute of Public Health (WIV-ISP), Brussels, Belgium; 3 Aspinall & Associates, Tisbury, United Kingdom; 4 Bristol University, Bristol, United Kingdom; 5 Resources for the Future, Washington, D.C., United States of America; 6 Technical University of Delft, Delft, Netherlands; 7 World Health Organization, Geneva, Switzerland; 8 University of Florida, Gainesville, Florida, United States of America; 9 National Institute for Public Health and the Environment, Bilthoven, Netherlands; 10 Utrecht University, Utrecht, Netherlands; 11 U.S. Centers for Disease Control and Prevention, Atlanta, Georgia, United States of America; 12 Gibb Epidemiology Consulting LLC, Arlington, Virginia, United States of America; 13 The Australian National University, Canberra, Australia; 14 Institute of Environmental Science and Research, Christchurch, New Zealand; 15 Université catholique de Louvain, Brussels, Belgium de Louvain, Brussels, Belgium; 16 University of Zurich, Zurich, Switzerland; 17 Technical University of Denmark, Lyngby, Denmark; University of Campinas, BRAZIL

## Abstract

**Background:**

Recently the World Health Organization, Foodborne Disease Burden Epidemiology Reference Group (FERG) estimated that 31 foodborne diseases (FBDs) resulted in over 600 million illnesses and 420,000 deaths worldwide in 2010. Knowing the relative role importance of different foods as exposure routes for key hazards is critical to preventing illness. This study reports the findings of a structured expert elicitation providing globally comparable food source attribution estimates for 11 major FBDs in each of 14 world subregions.

**Methods and findings:**

We used Cooke’s Classical Model to elicit and aggregate judgments of 73 international experts. Judgments were elicited from each expert individually and aggregated using both equal and performance weights. Performance weighted results are reported as they increased the informativeness of estimates, while retaining accuracy. We report measures of central tendency and uncertainty bounds on food source attribution estimate.

For some pathogens we see relatively consistent food source attribution estimates across subregions of the world; for others there is substantial regional variation. For example, for non-typhoidal salmonellosis, pork was of minor importance compared to eggs and poultry meat in the American and African subregions, whereas in the European and Western Pacific subregions the importance of these three food sources were quite similar. Our regional results broadly agree with estimates from earlier European and North American food source attribution research. As in prior food source attribution research, we find relatively wide uncertainty bounds around our median estimates.

**Conclusions:**

We present the first worldwide estimates of the proportion of specific foodborne diseases attributable to specific food exposure routes. While we find substantial uncertainty around central tendency estimates, we believe these estimates provide the best currently available basis on which to link FBDs and specific foods in many parts of the world, providing guidance for policy actions to control FBDs.

## Introduction

Recently the World Health Organization (WHO) established the Foodborne Disease Burden Epidemiology Reference Group (FERG) which estimated that 31 foodborne diseases resulted in over 600 million illnesses and 420,000 deaths worldwide in 2010 [1]. A quantitative understanding of the specific food exposures causing these foodborne diseases is critical to developing effective approaches to their prevention [2]. Food source attribution research partitions foodborne disease incidence among possible food exposure routes [3, 4]. This article presents median and mean food source attribution estimates and uncertainty intervals for 11 major foodborne illnesses for each of 14 global subregions. To our knowledge, this is the first set of globally comparable food attribution estimates and, for many lower income regions of the world, the first estimates attributing foodborne illnesses across the region’s food supply. This research was commissioned by the FERG’s Source Attribution Task Force (SATF) as part of its effort to develop estimates of the global burden of major foodborne diseases [5].

Several methods have been developed to attribute foodborne diseases to their food exposure sources including: use of microbiological subtyping combined with integrated surveillance; analysis of data from outbreak investigations; comparative exposure assessment; meta-analysis of case-control studies; and expert elicitation [3]. Many of these methods rely on surveillance or sampling data which are of limited availability, even in high income countries. An SATF commissioned evaluation of alternative food source attribution methods identified expert elicitation as the only feasible method for developing comparable estimates across low and high income regions of the world [6]. Expert elicitation has been used successfully in a wide range of applications where primary data is limited or absent, including foodborne disease source attribution [7, 8, 9, 10, 11, 12, 13].

This article is one of a set of related papers, many included in a PLOS collection [14] that report original research underlying the WHO Estimates of the Global Burden of Foodborne Diseases. The food source attribution estimates reported in this article are the results of an original global expert elicitation study designed specifically for FERG [15]. Hald et al. (2016) used different data from this elicitation to develop global estimates of the percentage of selected diseases caused by exposure through food and other major non-food exposure routes, such as water [16]. Aspinall et al. (2016) provide detail on the expert elicitation analysis method used in this study and on the performance of alternative approaches of aggregating experts’ judgments [17]. Estimated percentages reported in this article can be used to attribute *foodborne* disease incidence and burden to specific categories of food exposure in 14 global subregions.

## Materials and methods

### Study scope

The biology or chemistry of many hazards affects the primary exposure source or the relevance of specific foods as food exposures routes. This expert elicitation study was commissioned to provide food source attribution estimates for 11 hazards of the 31 foodborne hazards included in the WHO global burden of foodborne disease estimates. For 20 hazards, the SATF determined, based on review of the scientific literature, either that the likely food source could be determined with sufficient reliability without further research (e.g. *Vibrio vulnificus* infections linked to seafood), or that knowledge about the specific food exposures was assessed to be of little relevance for targeted disease prevention [15]. The latter group of hazards included pathogens with primarily a human reservoir (e.g. *S*. Typhi, *Shigella* spp. and *V*. *cholerae*), because the control of these pathogens is mainly a question of effective hygiene measures preventing spread from humans to food to humans. This is in contrast to the zoonoses like *Salmonella* spp. and *T*. *gondii*, where very specific intervention measures at the animal reservoir level may be key to successful prevention. The decision to include only 11 hazards in the final food attribution was also partly pragmatic in order to control the response burden placed the experts [1].

**Fig 1:**
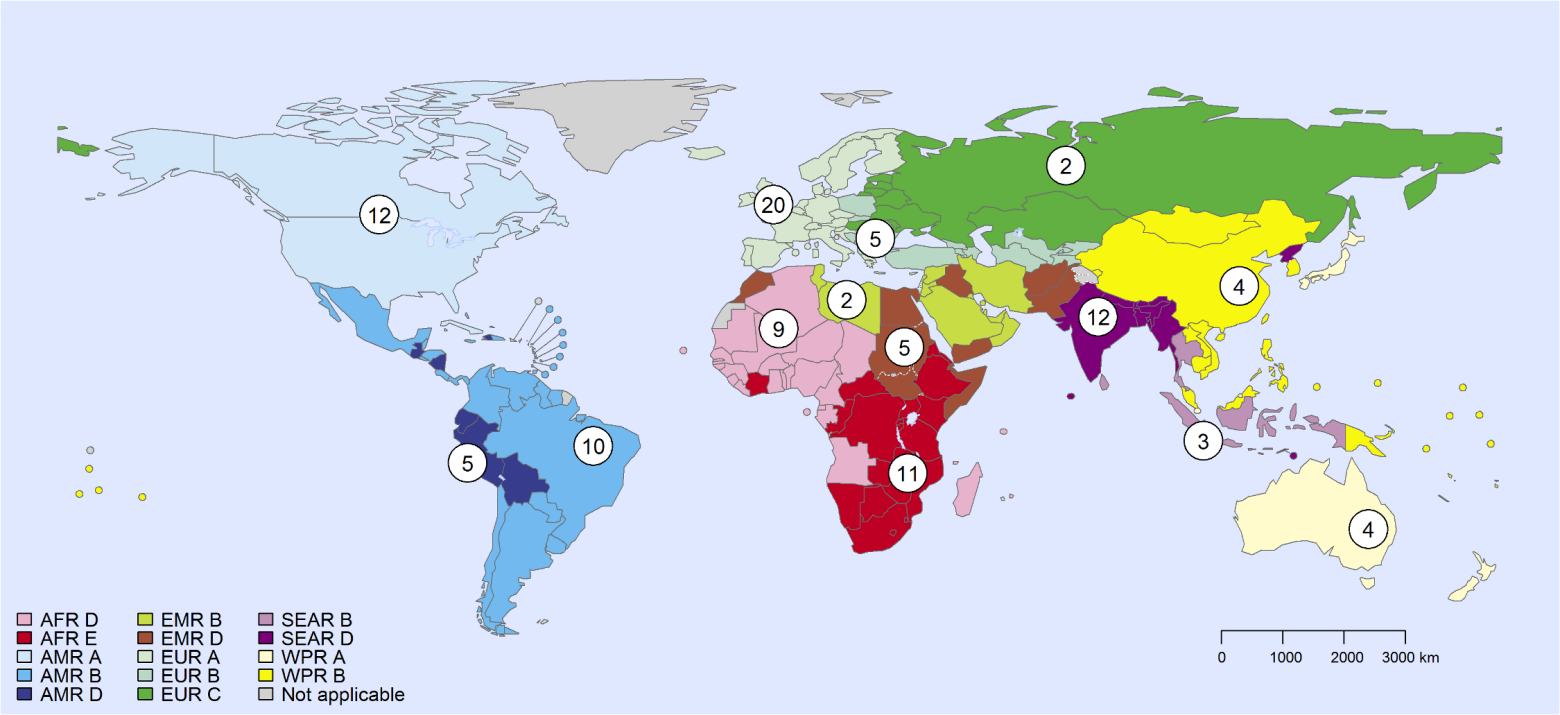
Global subregions and number of expert panelists with more than 3 years of work experience in the subregion. Subregions are defined on the basis of child and adult mortality as described by Ezzati et al. (2002) [18]. Stratum A: very low child and adult mortality; Stratum B: low child mortality and very low adult mortality; Stratum C: low child mortality and high adult mortality; Stratum D: high child and adult mortality; and Stratum E: high child mortality and very high adult mortality. AFR = Africa Region; AMR = Region of the Americas; EMR = Eastern Mediterranean Region; EUR = European Region; SEAR = South-East Asia Region; WPR = Western Pacific Region. Havelaar et al. 2015 provides a list of countries in each subregion [1]. The use of the term ‘subregion’ here and throughout the text does not identify an official grouping of WHO Member States, and the ‘subregions’ are not related to the six official WHO regions [16]. Values in circles indicate the number of experts in panels in each region.

Source attribution estimates were elicited separately for 14 global subregions used by FERG for the purpose of global burden of disease assessments. This subregional scheme is defined on the basis of WHO regions, and further subdivision based on mortality of children <5 years of age mortality (under-5) and of persons > = 5 years of age (adult mortality). Because it is based on all-cause mortality, this subregional classification reflects overall development levels and water and sanitation conditions, factors that also influence food handling and storage conditions (Fig 1) [18]. Subregions are classified on a scale ranging from A to E, with A having the lowest mortality rates and E the highest.

Foods were divided into 13 broad, mutually exclusive categories and an “Other food” category (Table 1). These food categories are similar to those used in other major food source attribution studies [11, 19, 20, 21]. Food categories reflect common contamination and consumption patterns. In order to reduce expert burden, food exposure routes deemed irrelevant or negligible for specific pathogens by SATF based on review of existing research were dropped (Table 1). A category “other food” was included in the elicitation to allow experts to indicate if any significant food category had been omitted.

**Table 1:** Food exposure routes elicited in WHO global burden of foodborne disease expert elicitation by pathogen*.

	Beef	Small ruminants’ meat	Dairy	Pigs’ Meat	Poultry Meat	Eggs	Vegetables	Fruit and Nuts	Grains and Beans	Oils and Sugar	Finfish	Shellfish	Seaweed
**Parasites**													
*Ascaris spp.*							x	x					
*Cryptosporidium spp.*			x				x	x					
*Echinoccocus granulosus*							x	x					
*Echinococcus multilocularis*							x	x					
*Entamoeba histolytica*							x	x					
*Giardia spp.*							x	x					
*Toxoplasma gondii*	x	x	x	x	x	x	x	x					
**Bacteria**													
*Brucella spp.*	x	x	x	x									
*Campylobacter spp.*	x	x	x	x	x		x	x					
*Non-typhoidal Salmonella spp.*	x	x	x	x	x	x	x	x	x	x	x	x	x
*Shiga-toxin producing E. coli*	x	x	x	x			x	x					

*The following hazards were included in the WHO global burden of foodborne disease estimates, but not in this elicitation of food source attribution estimates: Viruses: Norovirus, Hepatitis A; Bacteria: EPEC, ETEC, *Shigella* spp., *Vibrio cholerae*, *Listeria monocytogenes*, *Mycobacterium bovis tuberculosis*, *Salmonella* Paratyphi *A*, *Salmonella* Typhi; Parasites: *Taenia solium*, *Trichinella* spp., *Fasciola* spp., intestinal flukes, *Opisthorchis* spp., *Paragonimus* spp.; Chemicals and Toxins: aflatoxin, cassava cyanide, dioxin, lead.

**Fig 2:**
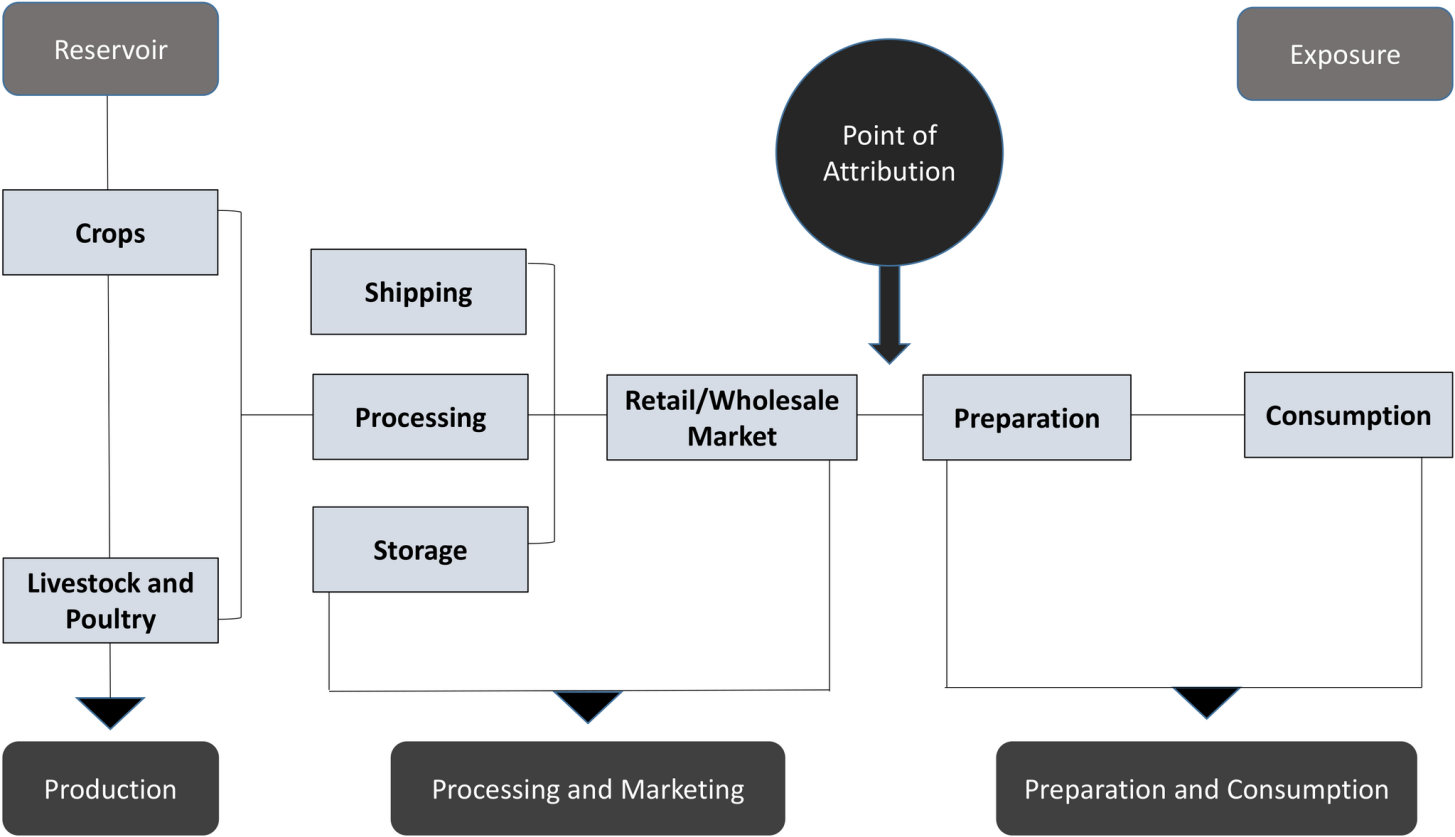
Attribution of foodborne illnesses to food contamination at the point it enters the place of final preparation for consumption.

Foodborne disease can be attributed to foods when consumed, to animal reservoirs, or as in this study, to foods contaminated at the point where they entered the place where they are prepared for final consumption (Fig 2). Hald et al. 2016 attributes illnesses to foodborne and other major exposure routes when the illness is caused by direct exposure through a given route, for example, ingested food or direct animal contact [16]. In the present study, foodborne illnesses are attributed to foods when they are caused by eating foods that were contaminated at the point that they entered the place where they were prepared for final consumption. For example, a person may become ill from eating a fresh green salad that was contaminated by a knife previously used to cut raw chicken. If the chicken had been contaminated with pathogens prior to entering the kitchen, we would attribute the illness to the chicken, not the lettuce.

We choose to use this point of attribution for this study for practical reasons. Different interventions are typically used in the supply chain than during food preparation and different people, including consumers, have responsibility for food safety during preparation than in the supply chain. In some countries different regulatory authorities govern the supply chain and food preparation. For example, in the United States, hygiene conditions in restaurants and institutional kitchens are typically governed by state and local authorities, while the processing, manufacture and transport of food that will be shipped across state borders is governed by federal law and national authorities. Modern food safety systems rely on both reduction of hazards in the food supply chain and hygiene and safe storage practices in places where food is prepared.

Information needed to understand how to improve food safety in the supply chain would be lost if attribution were made to the food that was contaminated at the point of consumption. Practices used to prevent contamination during food preparation are generally food hygiene and storage practices, which would affect the safety of any food in the kitchen or food preparation area. Interventions used further up the food supply chain can be more targeted and different from those used in the place of food preparation. For example, closure of fishing grounds is often used to prevent marketing of *V*. *vulnificus* contaminated shellfish; testing and culling of poultry flocks that test positive is used in some countries to reduce *Salmonella* prevalence. Appropriate or feasible supply chain interventions likely vary by geographic region and by level of economic development. The WHO and FAO have programs designed to help build food safety management capacity in the public and private sectors in lower income regions of the world [22].

### Analytical method

Gaps in data or primary research are a common problem confronting complex empirical modeling efforts like the estimation of the global burden of foodborne disease. In such cases, structured elicitation of expert judgments is increasingly used as a more transparent and consistent alternative to reliance on solely of model builders’ own judgments to ill in these gaps [6]. Research shows that individuals, including scientific experts, often exhibit systematically biased ways of assessing the likelihood of uncertain outcomes [23]. One expert might have a tendency to be overly confident in their own judgment, another might tend to over-estimate the likelihood of adverse outcomes. Expert elicitation methods use a variety of approaches for addressing these systematic tendencies [23]. The SATF chose to use Cooke’s Classical Model because of its reliance on transparent, quantitative performance measures which minimize the effects of individual expert biases, its successful use in prior food source attribution studies, its validation in numerous studies in many disciplines, and the feasibility of applying the method in a study of global scale [24, 25, 26, 27].

Cooke’s Classical Model uses calibration questions to measure an experts’ systematic tendencies in providing judgments under uncertainty [24]. Calibration questions require experts to provide judgments under conditions of uncertainty, that is, they ask experts to assign quantities and uncertainty bounds to outcomes whose values are not known to the expert at the time they answer the calibration question. For example, the calibration questions might ask about future values of time series on data in the expert’s field or about quantitative results from relevant research reviews which the expert cannot access when answering the calibration question. While the experts are not expected to know the precise values of the outcomes, each expert should be able to rationalize his or her understanding to provide median values located realistically close to the true values, with credible ranges that fittingly capture uncertainties about those values. Realization values for the calibration questions must become known to the study analysts within the timeframe of the study so that experts’ responses can be empirically evaluated.

Experts’ tendencies in forming judgments about the likelihood of uncertain events may vary by subject matter domain. For this reason, it is essential to use calibration questions that are related directly to the substantive subject matter domain of the target questions. In this study, because food attribution involves synthesis of a range of information, we developed calibration questions that reflect diverse factors that could inform experts’ judgments about food source attribution, including food supply and consumption patterns, water and sanitation conditions, and the incidence of diseases (Table 2). The full set of calibration questions used in this study is provided in S1 File.

**Table 2:** Example calibration questions used in WHO global burden of foodborne disease source attribution expert elicitation.

TOPIC	HAZARD	QUESTION
**Dietary Patterns and Food Supply**	All microbial hazards	Among all subregions in 2010, what was the proportion of regional vegetable supply (tonnes) that was imported rather than produced domestically in the subregion with the highest such percentage?
**Under-5 Mortality Rate**	*Brucella* spp., *Echinococcus* spp., intestinal protozoa, diarrheal pathogens	Based on WHO’s estimates, think of the country in the African Region that had the largest percentage point decrease from 2000 to 2010 in all-cause under-5 mortality that was due to diarrhea. What was that percentage point decrease?
**Disease Surveillance**	Enteric pathogens (developed subregions only)	What will be the rate per 100,000 population of laboratory confirmed human cases of campylobacteriosis in 2012 in all EU member states as reported in EFSA’s annual report?
**Systematic Review**	All microbial hazards	Fewtrell et al. (2005) conducted a systematic review and meta-analysis to compare the evidence of relative effectiveness of improvements in drinking water, sanitation facilities, and hygiene practices in less developed countries in reducing diarrheal illness. The meta-analysis of 5 studies was used to estimate the relative risk of diarrheal illness with and without multiple interventions. What was the estimated relative risk?

Analysis of experts’ performance on the calibration questions serves two purposes: 1) to evaluate the expert’s *statistical accuracy* when providing probability assessment under uncertainty (i.e., how reliably the expert’s credible interval responses contain the actual answers to the calibration questions, once they are known); and 2) to evaluate the *informativeness* of the uncertainty bounds the experts provide on the probability distributions elicited by the calibration questions (i.e., how narrow or concentrated are the distributions the expert provides). The statistical accuracy of responses is measured by the p-value at which the hypothesis that the expert’s probability assessments are statistically accurate would be falsely rejected. Informativeness is measured as Shannon relative information with respect to an analyst-defined background measure. Informativeness scores under the Classical Model are not absolute, but relative to the group of experts assessing the same variables [17, 25]. A more technical discussion of Cooke’s Classical Model is provided in S2 File.

Experts’ responses to calibration questions are used to develop performance weights used in aggregating experts’ judgments on target questions, i.e. the questions of substantive interest. *Performance weights* (PWs) for each expert jointly optimize the combined accuracy and information scores for the set of calibration questions. Typically, the Classical Model makes use of between eight and fifteen calibration questions. Past studies have shown that more calibration questions do not significantly affect the resulting PWs [24]. Unweighted aggregate judgments can also be estimated for a comparative benchmark. Aspinall *et al*. (2016) provides a more detailed discussion of how Cooke’s Classical Model was applied in the FERG source attribution research [17].

**Fig 3:**
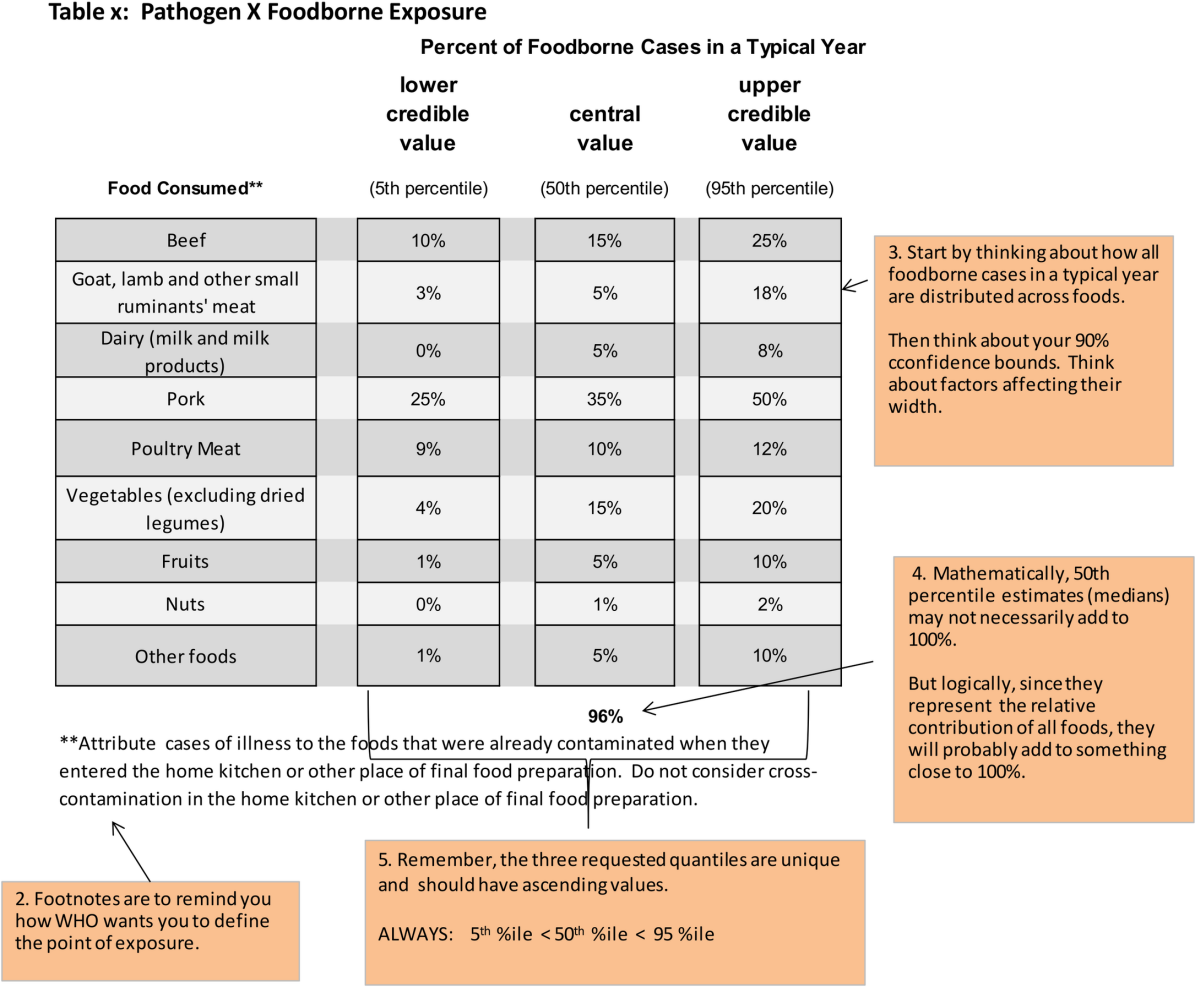
Example food source attribution questions provided in expert elicitation instructions.

Experts who participated in the study are located around the globe. In order to draw on a geographically disperse set of experts, elicitations were conducted via Skype or phone with written materials exchanged via email. During each elicitation session, a trained facilitator explained the overall study design, administered a relevant set of calibration questions, explained the food attribution task the expert was being asked to perform, and guided the expert through a hypothetical example set of target questions (Fig 3). Facilitators explained that the purpose of the calibration questions was to look for systematic tendencies in the expert’s judgments under uncertainty. Experts were asked not to consult any information sources when answering the calibration questions in order to ensure that they provided their judgments under conditions of uncertainty. The calibration questions were administered during the Skype call with the facilitator to provide additional assurance that the experts did not access additional information sources.

At the end of the SKYPE call, experts were provided tables, similar to that in Fig 3, to fill in for each pathogen/subregion pair for which their source attribution judgments were sought. Experts were asked to return the tables within 4 weeks. In these tables, experts were asked to provide their best judgments about the percentage of cases of a specific foodborne disease in a subregion in a typical year attributable to a specific food exposure route. In their SKYPE calls, facilitators explained how these attribution results would be used in estimating subregion-specific burden of foodborne disease estimates. These estimates related to the year 2010. As prevalence of hazards in food fluctuates over time and across subregions, experts were asked to consider their best judgments about the appropriate time window over which to “average” these fluctuations in providing attribution estimates. Experts were asked to provide a 90% credible interval around their median judgment. As is typically the case in expert judgment studies, these uncertainty bounds reflect all sources of uncertainty and variability, including the expert’s assessment of uncertainty related to the quality of existing data and research, and variability in the prevalence of the hazard on food and in the relationship of hazard prevalence to illness across the region and over time. For the target questions, unlike the calibration questions, experts were told they were free to consult any information source they felt relevant for completing their attribution judgment tables, including having discussions with colleagues. The elicitation instructions stressed, however, that ultimately the study sought their own individual judgments. Research on decision science and survey methodology has repeatedly shown that respondents, even expert respondents, tend to “anchor” on information provided in a survey or elicitation, biasing results [28, 29, 30, 31]. We did not provide experts with specific information for the elicitation, such as national food supply data which is readily available from the FAO website, because we were seeking their expert judgments based on the information they, as experts, believed relevant.

#### Identification of experts and structuring expert panels

The goal of an expert elicitation, like ours, is not to sample the entire population of relevant experts, but to obtain responses from an adequately sized panel of well-informed subject matter experts who are good at providing judgments under uncertainty. Experience with the Cooke method shows that panels of 8 to 15 experts are typically an optimal size [27]. In this study, experts were identified through iterative peer nomination [32]. Initial points of contact included leadership of relevant scientific societies, WHO regional offices, and leading epidemiologists and food scientists around the world working in fields related to foodborne disease. Final selection of expert panelists was made by the WHO in consultation with FERG. Attention was paid to avoiding conflicts of interests and to ensuring that the expertise of panel members adequately reflected the geographic scope of the task and the range of professional backgrounds and experience relevant to understanding foodborne disease transmission.

In our elicitation, many experts had experience working on the same hazards in multiple regions. As a result, expert panels were organized by hazard or classes of hazards rather than geographically. For all hazards except enteric pathogens, individual experts were asked to provide food attribution judgments for each of 14 subregions of the world. For enteric pathogens, separate panels were created for each subregion acknowledging that exposure to enteric pathogens is heavily influenced by the level of water and sanitation infrastructure, which differs significantly between subregions and that individual’s expertise relevant to food attribution of foodborne enteric disease also tends to be more regionally focused. Experts were free not to provide responses for specific subregions or pathogens and some experts served on multiple panels.

### Data analysis

Weights for individual experts were computed using Cooke’s Classical Model formulation with the TUDelft EXCALIBUR software [27]. Individuals’ calibration (statistical accuracy) and informativeness scores are multiplied together, with the products then jointly normalized over all experts in a panel to provide individual weights. These weights provide the basis for calculating an optimal performance-weighted-“decision-maker” (PW-DM) model. Statistical accuracy scores are p-values of falsely rejecting the hypothesis that the individual expert is statistically accurate. The informativeness scores, which indicate the narrowness or spread of experts’ uncertainty distributions, are slower functions than the statistical accuracy scores, so greater informativeness does not buy a higher calibration score at the expense of statistical accuracy–the latter predominates. When pooling experts’ responses to target questions, experts were positively weighted in the PW-DM if their statistical accuracy *p*-value was higher than a threshold determined by optimization [17].

Joint probability distributions across foods for each hazard/subregion pair were constructed using the normalized PW-DM models. For comparison, joint probability distributions were also developed using equal-weighted-“decision-maker” (EW-DM) models. In a final step, 10,000 random values from the marginal cumulative distributions were simulated. To ensure that the sampled attribution proportions summed to 100%, a ‘re-normalization’ step was applied per iteration, in which each random value was divided by the sum of all random values in that iteration, for each exposure pathway. The resulting 10,000 normalized random attribution proportions were then summarized by their median, mean and 95% uncertainty interval (2.5^th^ and 97.5^th^ percentiles) [17, S3 File].

## Results

### Expert performance

Of 100 experts invited by WHO to participate in the study, 78 accepted the invitation and completed calibration question interviews with facilitators; 73 returned completed responses to target questions. Analysis of performance on calibration questions showed that the number of responses was adequate to estimate statistically reliable aggregate models. In the entire WHO expert elicitation study, there were 112 hazard/subregion panels. Rounding the p-values to two digits, only one of resulting 112 PW DM models would be rejected at the 5% level and none would be rejected at the 1% level. While the statistical accuracy scores of the equal-weighted models are slightly higher than those of the PW-DM models, the corresponding information scores for the PW-DM models were better than those of the EW-DM models [17]. This ‘trade-off’ of informativeness against statistical accuracy, when applying equal weights and performance weights, is often seen in studies using the Classical Model [33, 34]. In our results, the combined performance score for performance-weighted models was higher than that of the equal-weighted models in 62% of cases. It was, therefore, decided to use the performance-weighted models in constructing the joint probability distributions for the food source attribution estimates, as long as statistical accuracy was acceptable [17].

### Food attribution results

**Fig 4:**
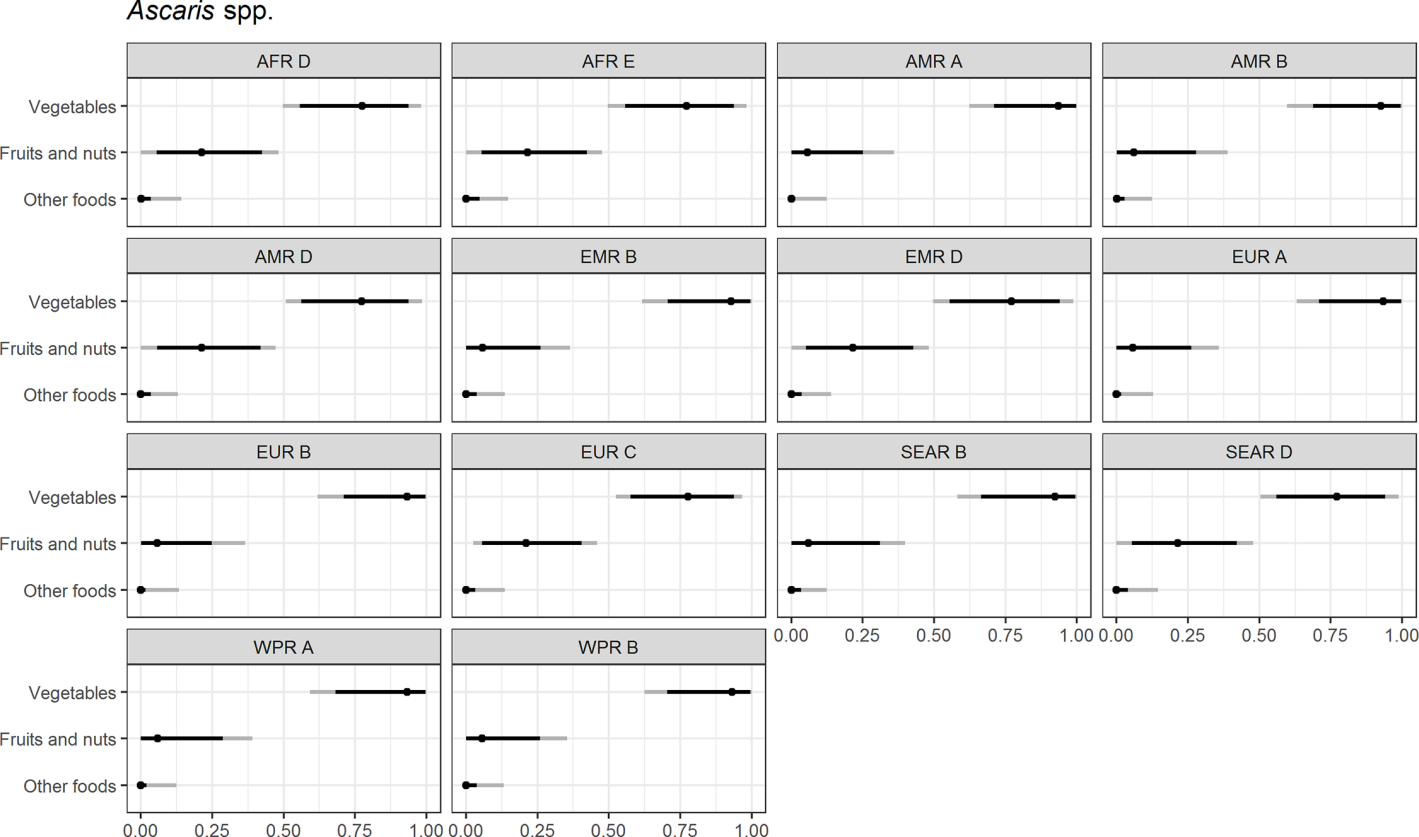
*Ascaris* spp. **Proportion of foodborne disease attributable to specific foods: Median estimate, and 90% and 95% uncertainty intervals by parasite and subregion.** “Small ruminants’ meat” was listed as “Goat, lamb and other small ruminants’ meat” in the expert elicitation instrument. The X-axis labels are percentages. The dot represents the median estimate; the dark black line, the 90% uncertainty interval; and the gray line the 95% uncertainty interval.

**Fig 5:**
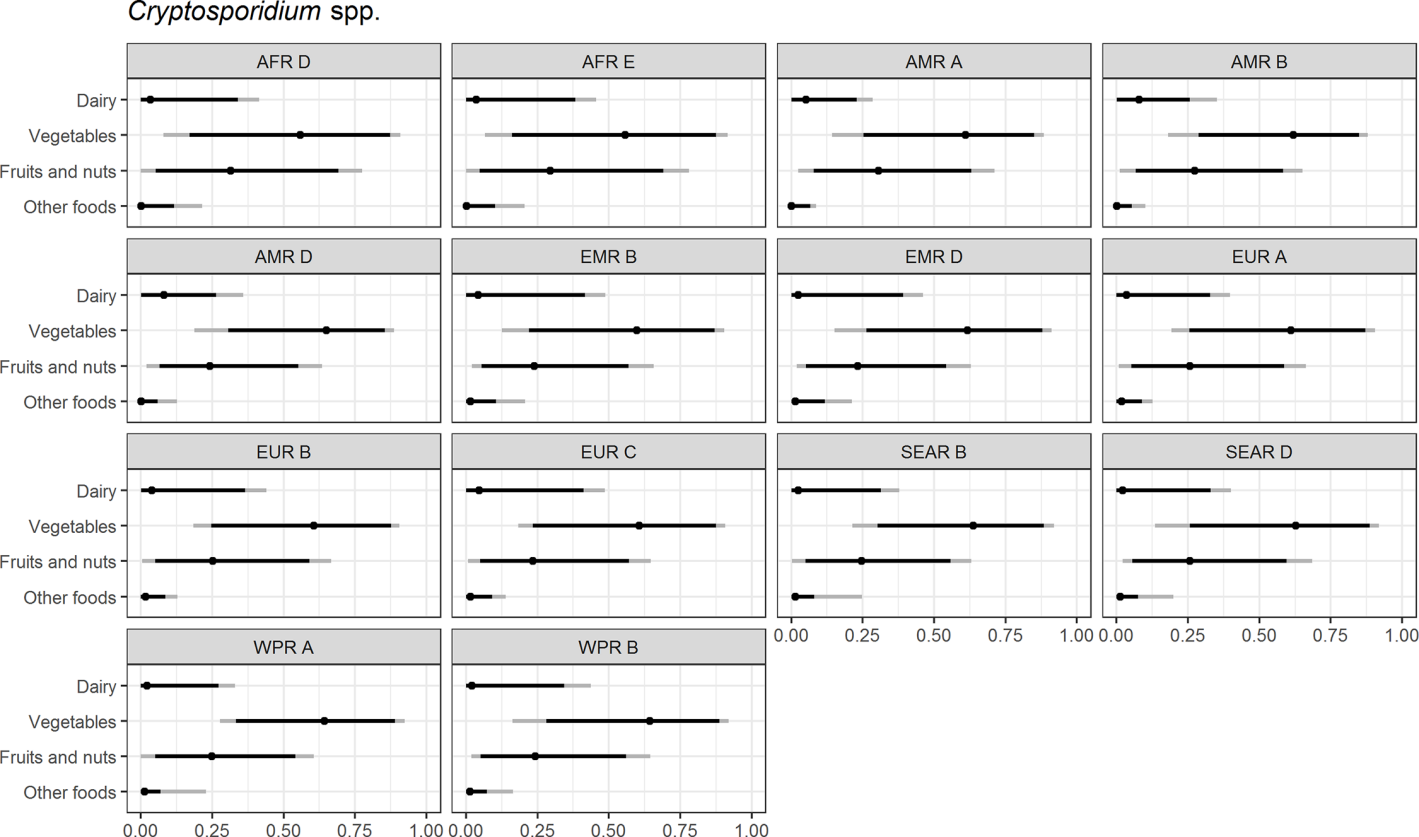
*Cryptosporidium* spp. **Proportion of foodborne disease attributable to specific foods: Median estimate, and 90% and 95% uncertainty intervals by parasite and subregion.** “Small ruminants’ meat” was listed as “Goat, lamb and other small ruminants’ meat” in the expert elicitation instrument. The X-axis labels are percentages. The dot represents the median estimate; the dark black line, the 90% uncertainty interval; and the gray line the 95% uncertainty interval.

**Fig 6:**
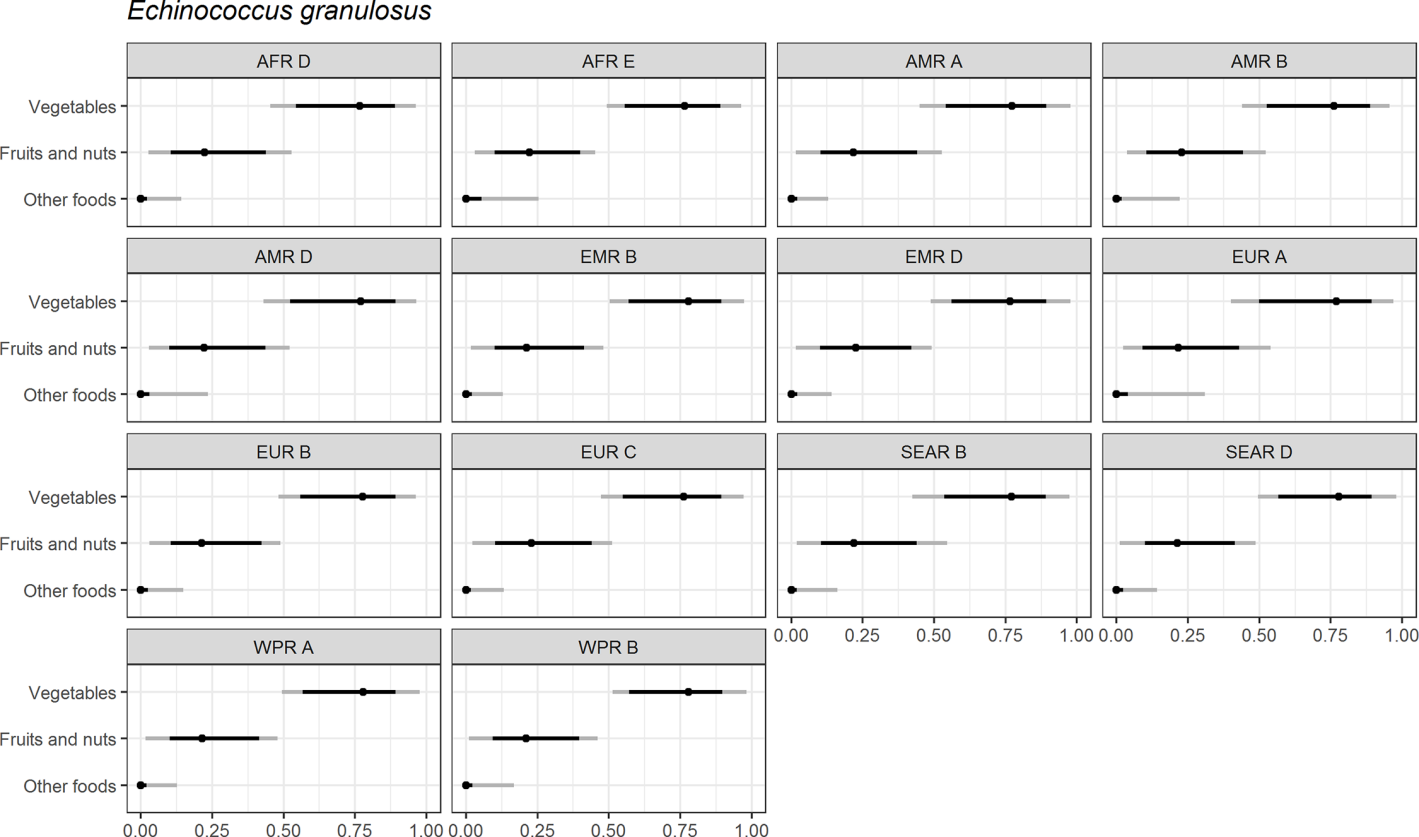
*Echinococcus granulosus*. **Proportion of foodborne disease attributable to specific foods: Median estimate, and 90% and 95% uncertainty intervals by parasite and subregion.** “Small ruminants’ meat” was listed as “Goat, lamb and other small ruminants’ meat” in the expert elicitation instrument. The X-axis labels are percentages. The dot represents the median estimate; the dark black line, the 90% uncertainty interval; and the gray line the 95% uncertainty interval.

**Fig 7:**
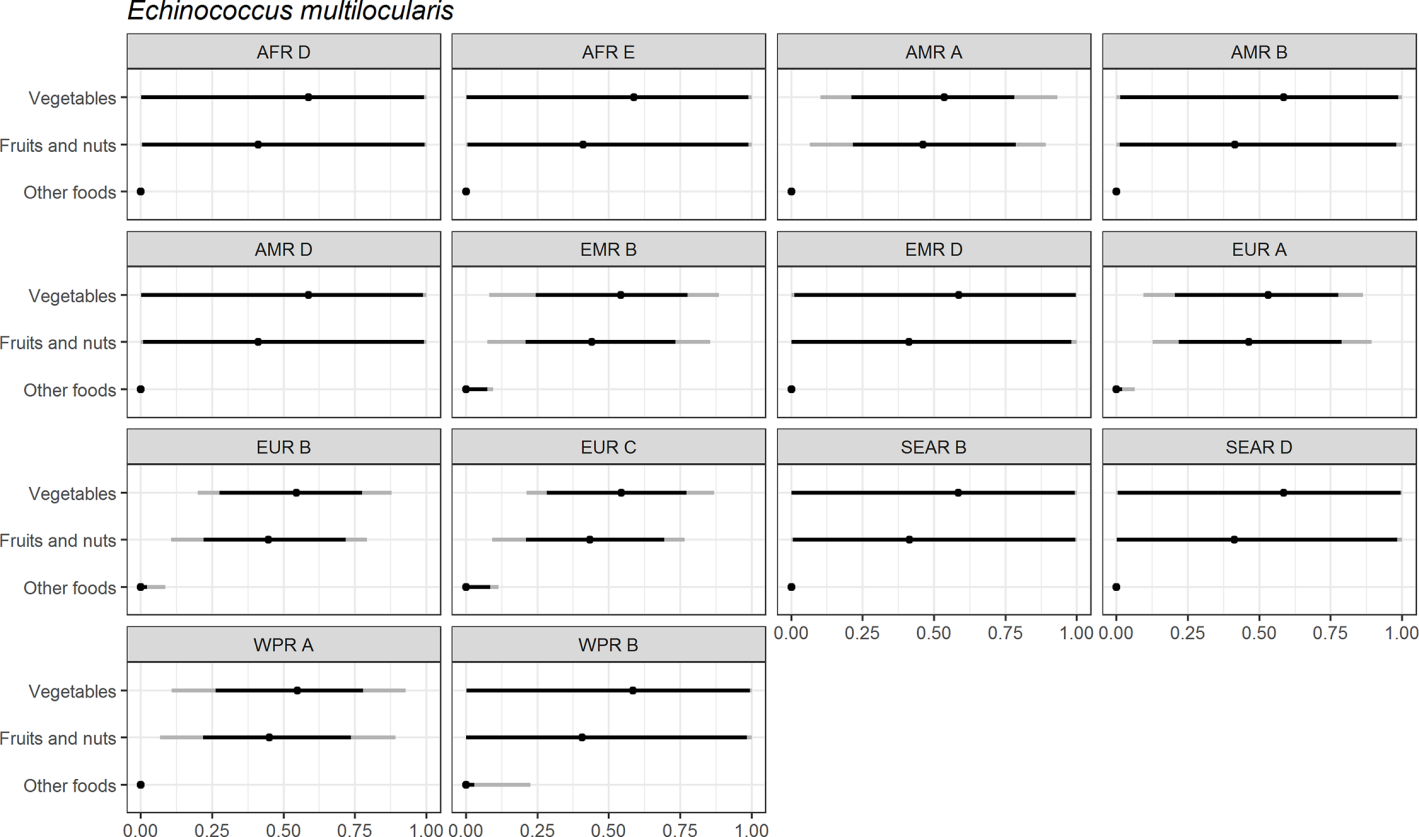
*Echinococcus multilocularis*. **Proportion of foodborne disease attributable to specific foods: Median estimate, and 90% and 95% uncertainty intervals by parasite and subregion.** “Small ruminants’ meat” was listed as “Goat, lamb and other small ruminants’ meat” in the expert elicitation instrument. The X-axis labels are percentages. The dot represents the median estimate; the dark black line, the 90% uncertainty interval; and the gray line the 95% uncertainty interval.

**Fig 8:**
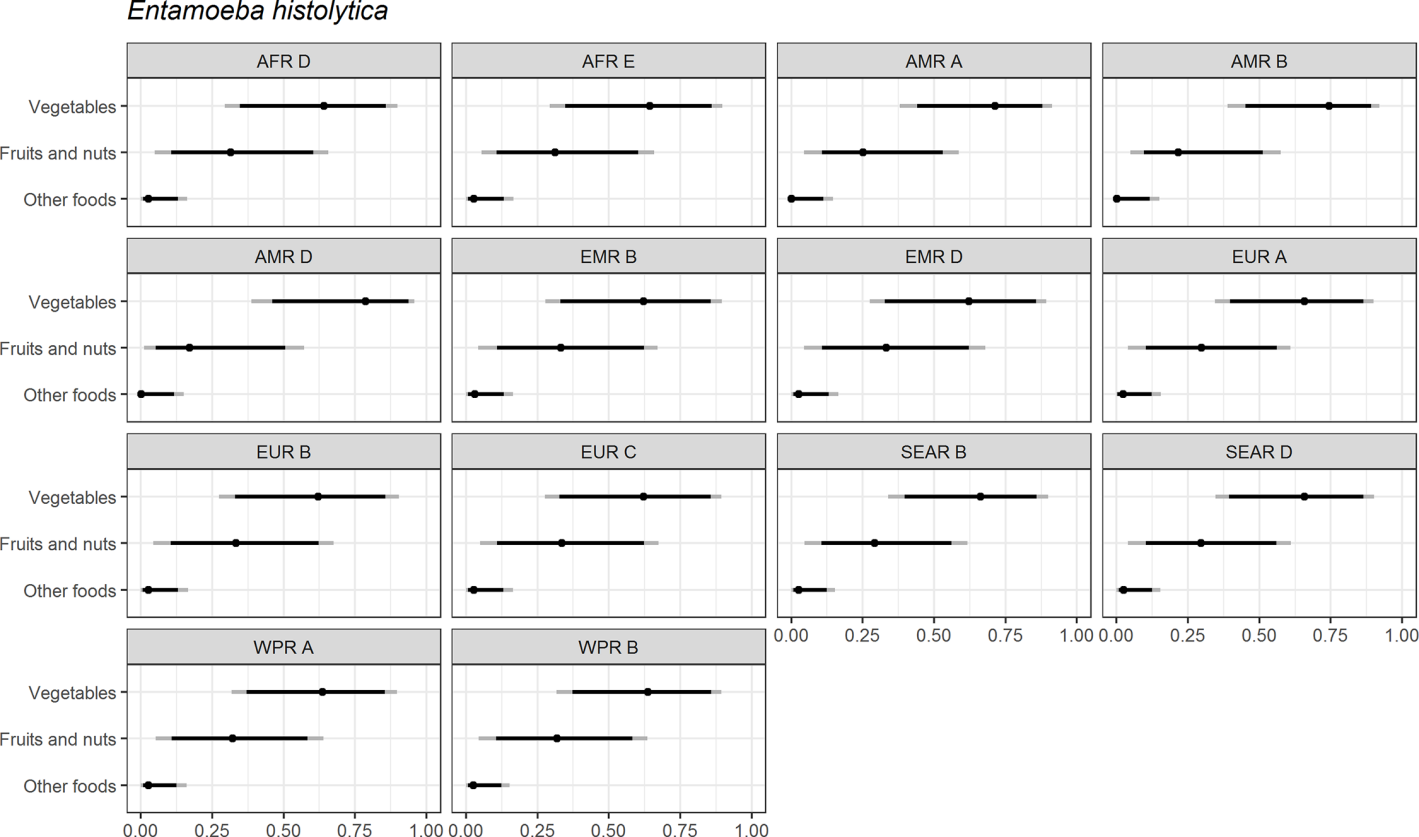
*Entamoeba histolytica*. **Proportion of foodborne disease attributable to specific foods: Median estimate, and 90% and 95% uncertainty intervals by parasite and subregion.** “Small ruminants’ meat” was listed as “Goat, lamb and other small ruminants’ meat” in the expert elicitation instrument. The X-axis labels are percentages. The dot represents the median estimate; the dark black line, the 90% uncertainty interval; and the gray line the 95% uncertainty interval.

**Fig 9:**
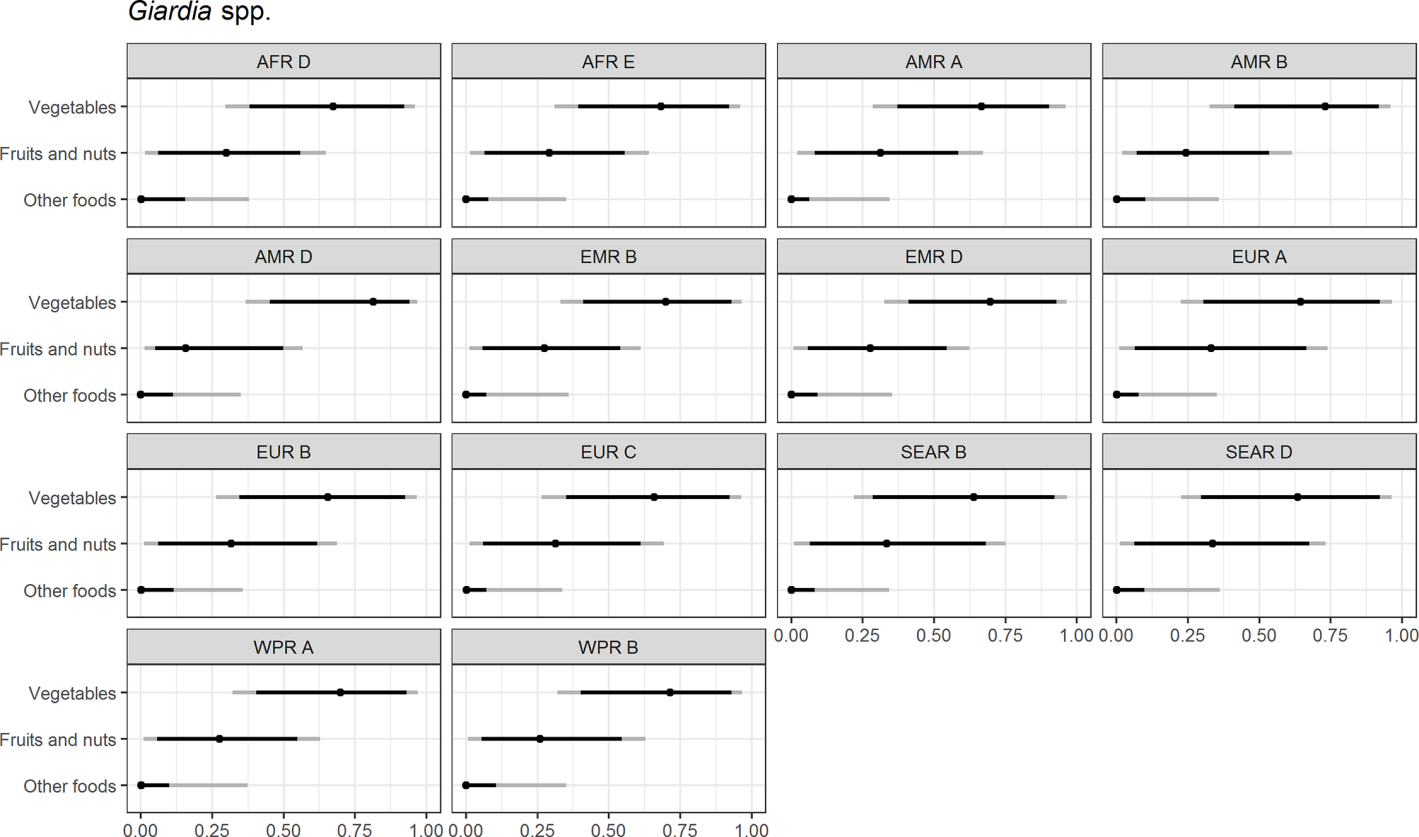
*Giardia* spp. **Proportion of foodborne disease attributable to specific foods: Median estimate, and 90% and 95% uncertainty intervals by parasite and subregion.** “Small ruminants’ meat” was listed as “Goat, lamb and other small ruminants’ meat” in the expert elicitation instrument. The X-axis labels are percentages. The dot represents the median estimate; the dark black line, the 90% uncertainty interval; and the gray line the 95% uncertainty interval.

**Fig 10:**
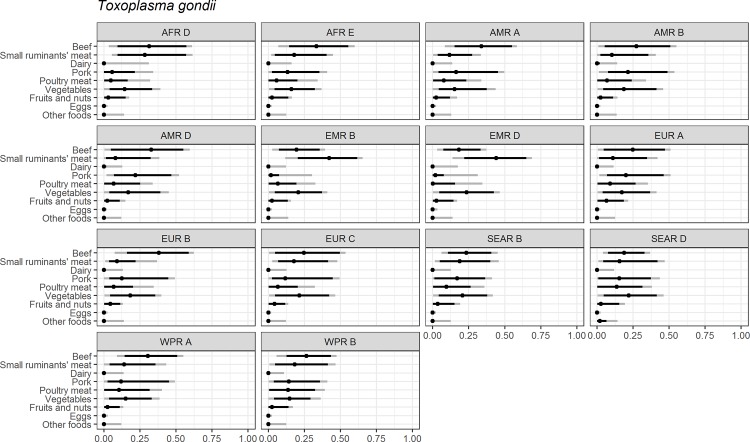
*Toxoplasma gondii*. **Proportion of foodborne disease attributable to specific foods: Median estimate, and 90% and 95% uncertainty intervals by parasite and subregion.** “Small ruminants’ meat” was listed as “Goat, lamb and other small ruminants’ meat” in the expert elicitation instrument. The X-axis labels are percentages. The dot represents the median estimate; the dark black line, the 90% uncertainty interval; and the gray line the 95% uncertainty interval.

**Fig 11:**
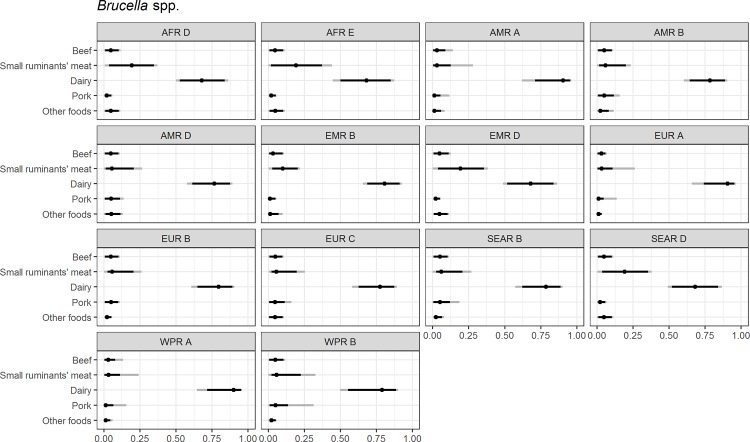
*Brucella* spp. **Proportion of foodborne disease attributable to specific foods: Median estimate, and 90% and 95% uncertainty intervals by bacteria and subregion.** “Small ruminants’ meat” was listed as “Goat, lamb and other small ruminants’ meat” in the expert elicitation instrument. The X-axis labels are percentages. The dot represents the median estimate; the dark black line, the 90% uncertainty interval; and the gray line the 95% uncertainty interval.

**Fig 12:**
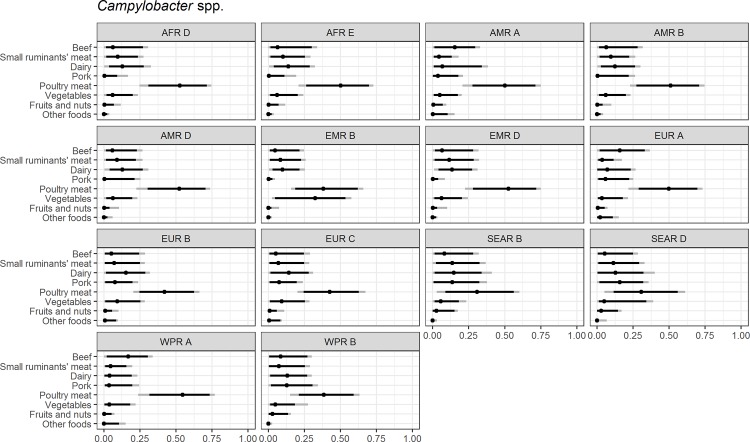
*Campylobacter* spp. **Proportion of foodborne disease attributable to specific foods: Median estimate, and 90% and 95% uncertainty intervals by bacteria and subregion.** “Small ruminants’ meat” was listed as “Goat, lamb and other small ruminants’ meat” in the expert elicitation instrument. The X-axis labels are percentages. The dot represents the median estimate; the dark black line, the 90% uncertainty interval; and the gray line the 95% uncertainty interval.

**Fig 13:**
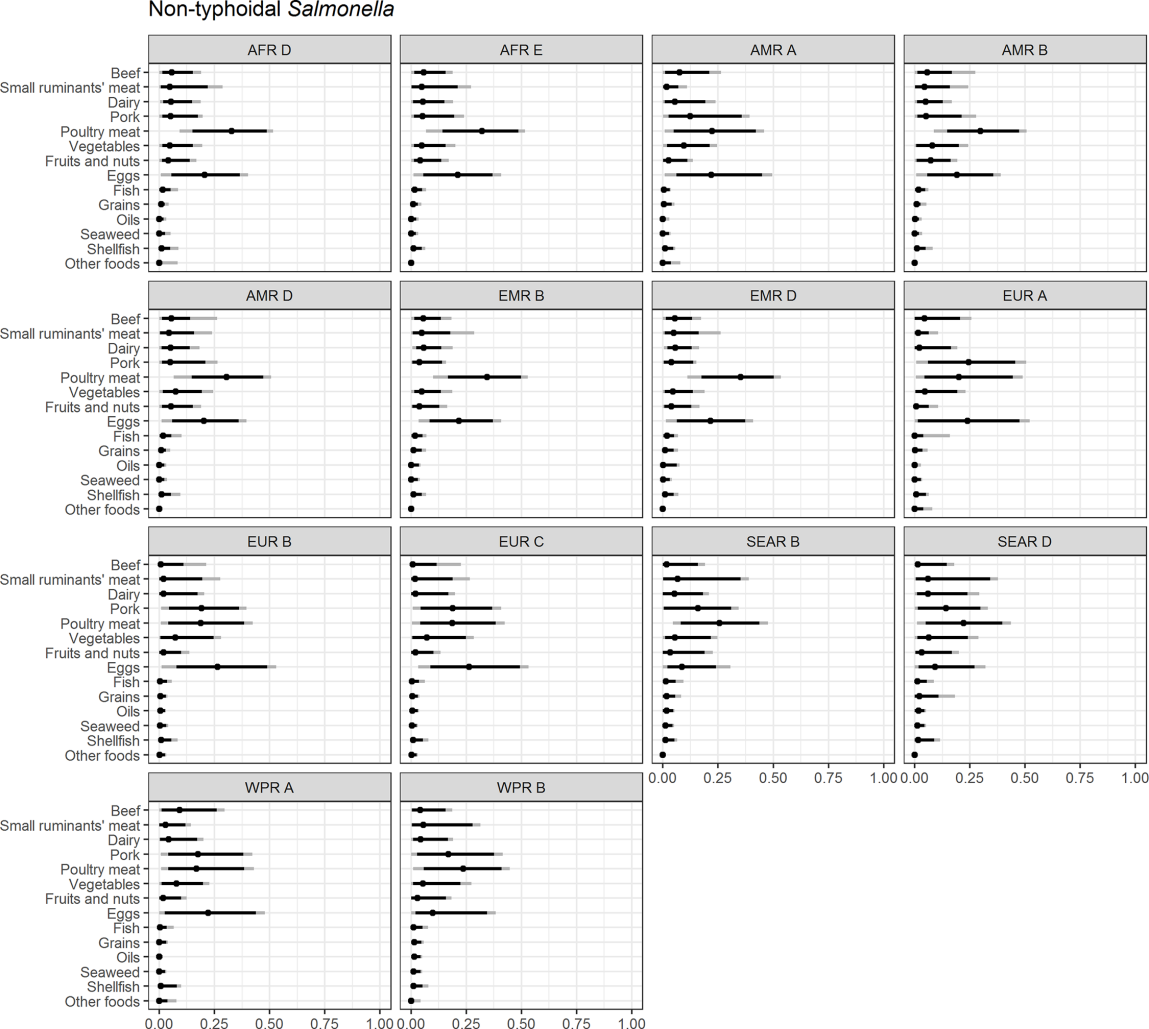
Non-typhoidal *Salmonella* spp. **Proportion of foodborne disease attributable to specific foods: Median estimate, and 90% and 95% uncertainty intervals by bacteria and subregion.** “Small ruminants’ meat” was listed as “Goat, lamb and other small ruminants’ meat” in the expert elicitation instrument. The X-axis labels are percentages. The dot represents the median estimate; the dark black line, the 90% uncertainty interval; and the gray line the 95% uncertainty interval.

**Fig 14:**
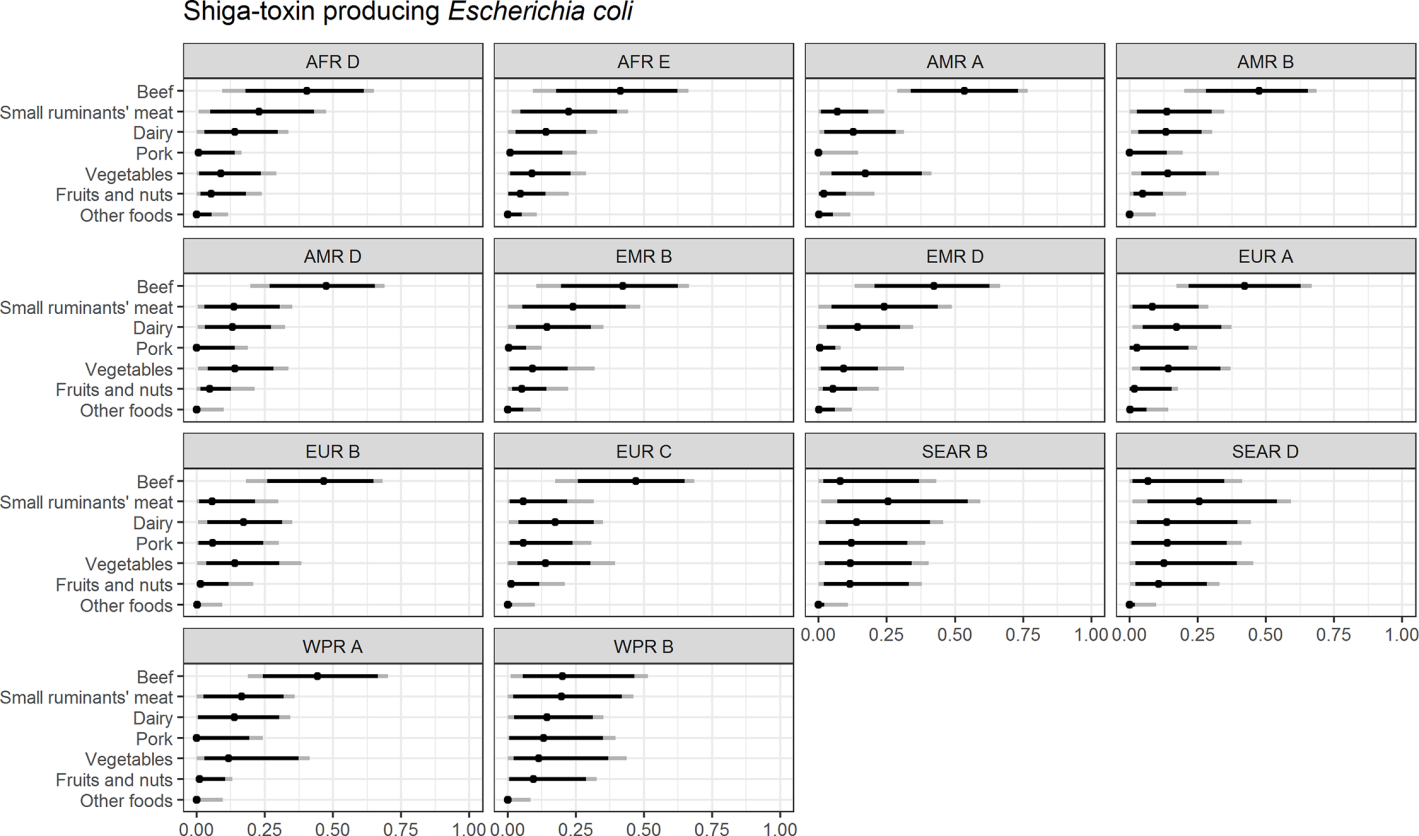
Shiga-toxin producing *Escherichia coli* (STEC). **Proportion of foodborne disease attributable to specific foods: Median estimate, and 90% and 95% uncertainty intervals by bacteria and subregion.** “Small ruminants’ meat” was listed as “Goat, lamb and other small ruminants’ meat” in the expert elicitation instrument. The X-axis labels are percentages. The dot represents the median estimate; the dark black line, the 90% uncertainty interval; and the gray line the 95% uncertainty interval.

Figs 4–14 present distributions of food attribution estimates by subregion. Numerical estimates for all food exposure routes are presented in S1 and S2 Tables. Median and uncertainty interval results are presented in Figs 4–14. Mean values were also modeled and are presented in S3 File. In general, mean and median values are highly correlated both by sub-region across foods, and by foods across subregion (Pearson correlation coefficients are generally above 0.98). The notable exception is that correlations for “other foods” across subregions are lower (.21 to .76), but the actual differences between mean values (generally in the range of less than 1 to 2 percent) and median values (generally approximately 0) are quite small from a practical perspective.

For the parasites, *Ascaris* spp., *Echinococcus granulosus*, *Echinococcus multilocularis*, *Entamoeba histolytica*, *Giardia* spp., and *Cryptosporidium* spp., vegetables are estimated to be the largest source of foodborne illness, accounting for 60% to 80% of illness in most subregions (Figs 4–10). The role of vegetables is estimated to be particularly important for *Ascaris* (over 90% for several subregions). Fruits and nuts are estimated to play a larger role for *E*. *multilocularis* (40% to 45%) than for other parasites (S1 Table). Regional variation in source attribution estimates is low for many of these parasites, in particular for *E*. *granulosus*. Among these parasites, *Ascaris* estimates have the greatest variability among subregions with fruits and nuts estimated to play less of a role in the A and B subregions than in other subregions. Fruits and nuts are also estimated to play less of a role in the American subregions for *E*. *histolytica* and in the mid- and high- mortality Region of the Americas (AMR) subregions (AMR B and AMR D) for *Giardia* spp. While the percent of foodborne illness attributed to “other foods” is very low for all of these parasites (0 to 3%), the wider uncertainty bounds for *Cryptosporidium* and *Giardia* suggest that the study design may have limited food options too much for these two parasites in many subregions. It is not possible to tell from our results whether the “other foods” that might be involved would be a limited or wide number of foods. Notably, both pathogens also have a lower percentage of total illness that is foodborne than the other parasites included in this study.

For foodborne toxoplasmosis, red meats (i.e. beef, small ruminants’ meat and pork) are estimated to cause 50% to 64% of foodborne cases in all regions, but the specific source of that exposure is estimated to vary markedly across subregions (Fig 10). Small ruminants’ meat is estimated to cause over 40% of foodborne toxoplasmosis in the Eastern Mediterranean Region (EMR) subregions. Beef is estimated to cause 30% to 40% of foodborne toxoplasmosis in the Africa Region (AFR) subregions, AMR A, AMR D, EUR B and Western Pacific Region (WPR) subregions. Pork is estimated to account for roughly 20% of foodborne toxoplasmosis in the AMR B, AMR D, and EUR A subregions. Vegetables are estimated to play a slightly larger role (21% to 23%) in the EUR subregions, and the South-East Asia Region (SEAR) subregions than in other subregions (14% to 19%). Eggs and dairy are not believed to contribute to foodborne toxoplasmosis (S1 Table).

For brucellosis, dairy and meat from small ruminants are estimated to cause 90% or more of foodborne brucellosis in all subregions, with 68% to 91% of this due to dairy exposure (S2 Table). Dairy is estimated to play a larger role in the low mortality (A) subregions than in other subregions and meat from small ruminants is estimated to play a greater role in the AFR subregions, EMR D and SEAR D (Fig 9).

For campylobacteriosis, the elicitation included many possible food exposure routes, including beef, dairy, fruits, small ruminant meat, poultry and vegetables. Most of these foods are estimated to account for less than 15% of illness (Fig 12). Poultry meat is estimated to cause 50% or more of foodborne campylobacteriosis in the “A” and the AFR, AMR and EMR subregions and over 40% in EUR B and EUR C. Beef, dairy, and pork are estimated to account for 15% to 20% of foodborne campylobacteriosis in the EUR A and EUR B subregions, the SEAR subregions and WPR A.

For foodborne salmonellosis, eggs are estimated to cause 15% or more of cases in all subregions except AMR A, and the SEAR and WPR subregions (Fig 13). Poultry is estimated to cause over 30% of foodborne salmonellosis in the AFR and EMR subregions. Pork is estimated to account for 15% to 20% of salmonellosis in the EUR, SEAR B and WPR B subregions. Patterns of other food exposures estimated to cause foodborne salmonellosis vary considerably by subregion, except that seaweed, shellfish, oils, and grains are not estimated to be substantial causes of foodborne salmonellosis illness in any subregions.

For foodborne Shiga toxin-producing *Escherichia coli* (STEC), beef is estimated to be the major cause of cases (40% to 55%) in all subregions except in the SEAR and WPR B subregions where meats from small ruminants are estimated to play the greatest role (20% to 26%) (Fig 14). Meats from small ruminants are also estimated to account for roughly 20% to 25% of illness from foodborne STEC in the AFR and EMR subregions. Dairy is estimated to cause roughly 15% of foodborne STEC illness in all subregions, but over 15% only in the EUR subregions. Vegetables are estimated to cause 17% of STEC illness in the AMR A subregion and 14% in the other AMR and EUR subregions (S2 Table).

The category “other food” was included to test for omission of important food categories. In 116 of the 154 pathogen/region pairs elicited, 0% of cases are estimated to be due to “other food” exposure. For no pathogen and subregion combination is this category estimated to contribute with more than 5% cases (S1 and S2 Tables). The largest estimates found were for *Brucella* spp. and *E*. *histolytica*, with 5% and 3% of cases respectively attributed to the category “other food” in several subregions. Uncertainty intervals around “other food” estimates are typically narrow, indicating that experts generally agreed with the SATF selection of food categories included in the elicitations, with the possible exceptions of *Cryptosporidium* and *Giardia* mentioned above.

## Discussion

Foodborne disease source attribution is a relatively new and rapidly developing area of research [3, 4, 11]. National or regional food source attribution studies have been conducted in high income countries of the AMR A and EUR A subregions. A number of these studies use microbial subtyping to attribute salmonellosis and campylobacteriosis to animal reservoirs, water and travel [see e.g., 35]. Ultimately, studies such as the one presented in this paper, which provides attribution estimates at the end of the supply chain, and studies that provide attribution estimates on farm and at other points in the supply chain are all useful in informing farm-to-table risk assessment. But because the point of attribution in these prior studies is on-farm instead of at the point where food enters the place of final preparation and usually is limited to farm animals or meats, it is difficult to compare the results of those studies to ours. Table 3–5 present results from studies that attribute illness to the point of exposure [8, 10, 11, 35]. While the results from these studies are not fully comparable because of differences in geographic coverage, method, food categorization and measure of central tendency reported, they do provide important context for our research.

**Table 3:** *Campylobacter* spp. Percent of foodborne illnesses attributed to specified food exposures from prior national studies and this study.

	*Campylobacter* spp.					
**Study**	**Davidson** **et al. (2011)**	**Hoffmann** **et al. (2007)**	**This Study**		**Havelaar** **et al. (2008)**	**This Study**
**Location**	Canada	U.S.	AMR A		Netherlands	EUR A
**Method**	EE	EE	EE		EE	EE
**Point of Attribution^ii^**	A	A	B		B	B
	Mean %	Mean %	Mean %		Median % (95% CI)	Median % (95% CI)
**Beef**	7.5	4.4	15.1			16 (0, 37)
**Beef and Lamb**					4 (0–17)	
**Small Ruminant Meats**			5.5			4 (0, 17)
**Beverages**	0.3	0.0			2 (0–4)	
**Bread and Bakery**	0.0	0.0				
**Grains**					2 (0–6)	
**Dairy**	9.2	7.8	12.2		9 (0–44)	7 (0, 27)
**Eggs**	4.5	2.6			3 (0–10)	
**Game**	1.8	2.0				
**Luncheon Meat**	1.4	0.9				
**Pork**	4.7	4.4	6.1		5 (0–19)	6 (0, 25)
**Poultry**	59.0	72.0	49.6		54 (17–86)	50 (22, 73)
**Produce (Fruits and Vegetables)**	6.1	5.2			5 (0–25)	
**Fruits and Nuts**			2			1 (0, 7)
**Vegetables**			6.8			3 (0, 21)
**Fish/ Shellfish**	0.8	0.8			7 (0–27)	
**Other Foods**	1.0	0.0	2.8			2 (0, 15)
**Composite Foods**					3 (0–10)	
**Human And Animal Exposure**					5 (0–18)	

EE = Expert elicitation; OB = Outbreak case analysis. A = Point of consumption, disregard cross contamination, water and travel; B = point of entry to the place of final food preparation, disregard water and travel; C = variable

**Table 4:** *Cryptosporidium* spp. Percent of foodborne illnesses attributed to specified food exposures from prior national studies and this study*.

	*CRYPTOSPORIDIUM SPP*.^*i*^						
**STUDY**	**Davidson** **et al. (2011)**	**Hoffmann** **et al. (2007)**	**Painter** **et al. (2013)**	**This study**		**Havelaar** **et al. (2008)**	**This study**
**LOCATION**	Canada	U.S.	U.S.	AMR A		Netherlands	EUR A
**METHOD**	EE	EE	OB	EE		EE	EE
**POINT OF ATTRIBUTION**	A	A	C	B		B	B
	Mean %	Mean %	Mean %	Mean %		Median % (95% CI)	Median % (95% CI)
**BEEF**	13.3	7.4	0.0				
**BEEF AND LAMB**						26 (24–56)	
**SMALL RUMINANT MEATS**							
**BEVERAGES**	13.1	9.0				3 (0–4)	
**BREAD AND BAKERY**	0.0	0.3					
**GRAINS**						(0–0)	
**GRAINS & BEANS**			0.0				
**DAIRY**	4.4	5.8	0.0	7.4		9 (6–20)	4 (0, 40)
**EGGS**	0.0	0.3	0.0			3 (0–5)	
**GAME**	2.9	5.4	0.0				
**LUNCHEON MEAT**	0.3	1.4	0.0				
**PORK**	2.5	2.0	0.0			4 (2–9)	
**POULTRY**	0.8	1.2	0.0			3 (1–5)	
**PRODUCE (FRUITS AND VEGETABLES)**	36.8	59.5				21 (20–38)	
**FRUITS AND NUTS**			100.0	32			26 (1, 66)
**VEGETABLES**				58.9			61 (19, 91)
**FISH/ SHELLFISH**	2.1	7.7	0.0			22 (21–38)	
**OTHER FOODS**	10.6			1.7			2 (0, 13)
**COMPOSITE FOODS**						3 (0–5)	
**HUMAN AND ANIMAL EXPOSURE**						6(4–11)	

*This study elicited values for *Cryptosporidium* spp., prior national studies elicited values for *C*. *parvum*. EE = Expert elicitation; OB = Outbreak case analysis. A = Point of consumption, disregard cross contamination, water and travel; B = point of entry to the place of final food preparation, disregard water and travel; C = variable

**Table 5:** Non-typhoidal *Salmonella* spp. Percent of foodborne illnesses attributed to specified food exposures from prior national studies and this study.

	NON-TYPHOIDAL *SALMONELLA* SPP.						
**STUDY**	**Davidson** **et al. (2011)**	**Hoffmann** **et al. (2007)**	**Painter** **et al. (2013)**	**This study**		**Havelaar** **et al. (2008)**	**This study**
**LOCATION**	Canada	U.S.	U.S.	AMR A		Netherlands	EUR A
**METHOD**	EE	EE	OB			EE	EE
**POINT OF ATTRIBUTION**	A	A	C	B		B	
	Mean %	Mean %	Mean %	Mean %		Median % (95% CI)	Median % (95% CI)
**BEEF**	5.8	10.9	7.3	8.8			4 (0, 26)
**BEEF AND LAMB**						13 (5–28)	
**SMALL RUMINANT MEATS**				2.5			2 (0, 11)
**BEVERAGES**	0.8	1.7				3 (0–9)	
**BREAD AND BAKERY**	2.1	0.3					
**GRAINS**				1.2		4 (0–12)	0 (0, 6)
**GRAINS & BEANS**			2.9				
**DAIRY**	7.1	7.3	7.2	7.2		7 (0–25)	2 (0, 19)
**EGGS**	21.0	21.8	14.8	23.3		22 (11–54)	24 (0, 52)
**GAME**	1.5	1.6	0.4				
**LUNCHEON MEAT**	4.8	1.9					
**OIL**				0.4			
**PORK**	7.2	5.7	6.2	15.5		14 (6–36)	24 (1, 50)
**POULTRY**	34.2	35.1	19.0	22.6		15 (5–47)	20 (1, 49)
**BROILERS**							
**TURKEYS**							
**PRODUCE (FRUITS AND VEGETABLES)**	17.8	11.7				6 (0–20)	
**FRUITS AND NUTS**			13.0	4			1 (0, 11)
**VEGETABLES**			28.0	10.1			5 (0, 23)
**FISH/ SHELLFISH**	1.6	2.0	1.1			6 (0–20)	
**FISH**				1			0 (0,16)
**SHELLFISH**				1.6			1 (0, 6)
**SEAWEED**				0.7			
**OTHER FOODS**	1.9			1.0			0 (0, 8)
**COMPOSITE FOODS**						6 (0–18)	
**HUMAN AND ANIMAL EXPOSURE**						6 (0–18)	
**TRAVEL**							
**UNKNOWN**							

EE = Expert elicitation; OB = Outbreak case analysis. A = Point of consumption, disregard cross contamination, water and travel; B = point of entry to the place of final food preparation, disregard water and travel; C = variable

In the AMR A subregion, with a few notable exceptions, our findings are in general agreement with earlier studies. This is to be expected if our method is working well and provides external validation of the approach we use in this study. For foodborne *Cryptosporidium* illnesses, all studies rank produce as the leading exposure route. Our study and the Painter et al. (2013) study using outbreak data find produce to be virtually the sole exposure route, while past expert elicitations in the U.S. and Canada see additional foods as playing a minor role (Table 4) [36]. For foodborne STEC O157, all expert elicitation studies find beef as the primary exposure route, with produce as the second most important route of foodborne exposure. Painter et al.’s 2013 analysis of outbreak data shows produce and beef as of roughly equivalent importance as foodborne STEC exposure routes [36]. For foodborne salmonellosis, all studies find poultry, eggs and produce as important sources of exposure, but the degree of importance differs across studies. Prior expert elicitation studies found poultry caused a third of foodborne salmonellosis and eggs about 20%. Our study estimates that poultry and eggs each cause about 20% of foodborne salmonellosis. In contrast, outbreak analysis ranked produce as the leading cause of foodborne salmonellosis followed by poultry and then eggs. For foodborne campylobacteriosis, past research indicates that outbreak data does not provide a good guide to exposures causing sporadic illnesses [21, 37, 38]. Further, most foodborne campylobacteriosis is likely sporadic [39]. All expert elicitation studies rank poultry meat as the leading exposure route (50 to 72%) for foodborne campylobacteriosis. Prior studies found 4 to 8% of foodborne campylobacteriosis as due to beef, 8 to 9% due to dairy and 5.5 to 7.7% distributed across eggs, game and luncheon meat; our study finds 15% of illness due to beef and 12% due to dairy (Table 3).

In the EUR A subregion, the only comparable study for most pathogens is an expert elicitation of food source attribution in the Netherlands using the same expert elicitation method as is used in this study, though with equal weighted expert models [10]. This Dutch study includes “human and animal exposure” in addition to food exposure routes, but as this non-food route usually accounts for only 5 to 6% of illness it is possible to compare their results to ours in terms of rankings and rough numerical comparisons.

Our attribution of foodborne Cryptosporidiosis to food exposures for the EUR A subregion differs substantially from those in the Dutch study (Table 4). The Dutch study assigned 26% of *Cryptosporidium* exposure (including foodborne, animal and human exposure routes) to beef and lamb, 22% to fish and shellfish, and 21% to produce [10]. Our estimates attribute 87% of foodborne Cryptosporidiosis to produce. This difference may be due to a relatively poor fit for our *Cryptosporidium* attribution model. For STEC O157, both the Dutch study and our study find beef, or “beef and lamb,” to be the primary food exposure route for foodborne illness, followed by dairy and produce [10]. Both studies indicate that poultry causes roughly half of foodborne campylobacteriosis, but disagree about the importance of other foods (Table 3). Our study identifies beef as the second leading food exposure route for campylobacteriosis in the EUR A subregion, while the Dutch study results showed non-poultry foodborne exposure spread over a wide range of other foods [9]. For foodborne salmonellosis, both studies estimate that 20 to 25% of illness is caused by eggs with pork and poultry playing important secondary roles (Table 5). But while the Dutch study found “beef and lamb” as being equally important as pork and poultry meat as exposure routes for foodborne salmonellosis, our study results show foods other than eggs, pork and poultry playing a very small role in the EUR A subregion (Table 5).

Uncertainty bounds around our estimates are generally wide. Wide uncertainty bounds were expected in many parts of the world where data that could be used to estimate food source attribution are sparse or non-existent. In these circumstances, expert judgments must be informed by basic knowledge of the biology of hazards, of food production and handling practices, food consumption patterns, and an understanding of the influences of water quality and sanitation generally, rather than be based on data and observed outcomes. But uncertainty intervals are also wide in AMR A and EUR A, where data and research is strongest and which have been centers of food attribution research. While there are many areas of agreement across these studies, the wide uncertainty bounds and the areas of disagreement show how difficult it is to estimate the role of specific foods in foodborne illness exposure, even in settings with the best available disease surveillance and sampling. Still, generally broad agreement about the primary food exposure categories across studies is a positive indication that our food attribution provides broad guidance about the relative importance of different foods in causing foodborne diseases.

Looking across pathogens and subregions, the results reported are generally consistent with what is known of pathogen ecology and food patterns. For example, it is unsurprising that dairy accounts for roughly 70% to 90% of foodborne brucellosis at the subregion level with variation that is consistent with dairy consumption patterns (S2 Table). But in some cases, we do see results that seem surprising given knowledge about food consumption patterns. While the median estimate that pork causes 2% of toxoplasmosis in EMR countries may seem reasonable, the uncertainty interval of 0% to roughly 30% may seem high for a predominantly Muslim region. Similarly, the estimate for the role of beef in causing toxoplasmosis may seem high in SEAR D, 19%, which has a large Hindu and Buddhist population.

Finally, it is important to bear in mind that expert elicitation should not replace use of ‘hard’ data, however it is useful where such data is unavailable or has significant limitations. In these situations, studies have conventionally relied on the judgments of study authors or modelers whose uncertainty judgments may reflect specific experience or specialism bias. Formal structured elicitation of judgments from a panel of multiple experts provides a systematic, transparent and auditable alternative. This is exactly the situation that was found in efforts to estimate the global burden of foodborne disease and attribution of this burden to specific food exposures. A challenge for national and global health researchers is to develop “hard” data that can obviate the need for use of expert judgment elicitations or, less transparently, individual modeler judgment [3].

## Conclusions

This study uses a structured expert elicitation to develop estimates of the role of specific foods as pathways contributing to foodborne disease from each of eleven pathogen hazards for 14 subregions of the world. Our results broadly agree with, and complement, estimates from earlier European and North American food source attribution research. For some parasites and *Brucella* spp., which have global panels, we see substantial regional variation in food source attribution, e.g., *Giardia* spp., *Entamoeba histolytica*, *Ascaris* spp., and *Toxoplasma gondii*, while for other parasites we see significant similarity across subregions, e.g., *Echinococcus granulosus*, and *Echinococcus multilocularis*. For the enteric pathogens, subregional panels are necessary due to the importance of water and sanitation conditions, which vary from one subregion to another, and due to regionally-specialized expertise. While we do see regional variation in the food source attribution estimates for enteric pathogens, factors in addition to water and sanitation seem to be at play. Variation in relative uncertainty bounds provide one piece of evidence on the relative strength of understanding of food source attribution by food, pathogen and subregion.

To our knowledge, these estimates provide the best currently available basis with which to assess the link between foodborne illnesses and foods in many parts of the world. Even with wide uncertainty bounds, these results provide important decision support information for public health institutions and donors around the world. Our results present food attribution estimates at the end of food supply chains, just prior to final food preparation. As such, they can provide a useful input into farm-to-fork risk assessments, particularly for lower income regions of the work. This research, and the larger project estimating the global burden of foodborne disease of which it is a part, provide a foundation for national and international policy efforts to build stronger risk-based food safety systems, explore targeted intervention options, and initiate research initiative to bridge the identified knowledge gaps. As our uncertainty results indicate, these efforts need to include development of stronger disease surveillance systems, particularly in lower income countries.

## Supporting information

S1 Table Median proportion of total foodborne cases attributed to exposure to specified foods, by subregions for selected parasites. Median proportion of total foodborne cases attributed to exposure to specified foods, by subregions for selected parasites.
(PDF)

S2 Table Median proportion of total foodborne cases attributed to exposure to specified foods, by subregions for selected bacteria. Median proportion of total foodborne cases attributed to exposure to specified foods, by subregions for selected bacteria.
(PDF)

S1 File Calibration (seed) questions for microbiological hazards. Calibration questions for microbiological hazards.
(XLSX)

S2 File Supporting technical information on the Cooke’s Classical Model. Supporting technical information on the Cooke’s Classical Model.
(PDF)

S3 File Percent of foodborne incidence attributable to food-specific exposures (means and medians). Percent of foodborne incidence attributable to food-specific exposures (means and medians).
(PDF)

S4 File Data file including anonymized responses from all experts. Data file including anonymized responses from all experts on food source attribution for the WHO FERG expert elicitation.
(XLSX)

S5 File Example elicitation instrument: Enteric pathogens food source attribution for the WHO FERG expert elicitation on food source attribution. Example elicitation instrument: Enteric pathogens food source attribution for the WHO FERG expert elicitation on food source attribution.
(XLS)

## Abbreviations

FERG: Foodborne Disease Burden Epidemiology Reference Group
FBD: Foodborne disease
SATF: FERG Source Attribution Task Force
DOI: Declaration of Interests
SEJ: Structured Expert Judgment
WHO: World Health Organization
PWs: performance weights
