# Supplementary material for: Attribution of global foodborne disease to specific foods: Findings from a World Health Organization structured expert elicitation
Source: PLoS One. 2017 Sep 14;12(9):e0183641. doi: 10.1371/journal.pone.0183641 (PMC5598938; doi:10.1371/journal.pone.0183641)
Supplement: S1 Table — Median proportion of total foodborne cases attributed to exposure to specified foods, by subregions for selected parasites. (PDF) [file pone.0183641.s001.pdf]

S1 Table. Median proportion of total foodborne cases attributed to exposure to specified foods, by subregions for selected parasites.

	AFR D	AFR E	AMR A	AMR B	AMR D	EMR B	EMR D	EUR A	EUR B	EUR C	SEAR B	SEAR D	WPR A	WPR B
Number of Experts	9	11	12	10	5	2	5	20	5	2	3	12	4	3
Ascaris spp.														
Percent of total disease that is foodborne*	0.38 (0.10-0.66)	0.38 (0.07-0.67)	0.83 (0.43-0.97)	0.55 (0.17-0.75)	0.37 (0.07-0.68)	0.55 (0.15-0.77)	0.55 (0.18-0.75)	0.85 (0.47-0.97)	0.55 (0.13-0.76)	0.55 (0.14-0.76)	0.54 (0.18-0.75)	0.39 (0.11-0.68)	0.85 (0.47-0.97)	0.54 (0.16-0.77)
Percent of foodborne disease attributed to:														
Fruits	0.21 (0.00-0.48)	0.21 (0.00-0.48)	0.06 (0.00-0.36)	0.06 (0.00-0.39)	0.21 (0.00-0.47)	0.06 (0.00-0.36)	0.22 (0.00-0.48)	0.06 (0.00-0.36)	0.06 (0.00-0.37)	0.21 (0.02-0.46)	0.06 (0.00-0.40)	0.21 (0.00-0.48)	0.06 (0.00-0.39)	0.06 (0.00-0.35)
Vegetables	0.77 (0.50-0.98)	0.77 (0.50-0.98)	0.94 (0.62-1.00)	0.93 (0.60-1.00)	0.77 (0.51-0.98)	0.93 (0.62-1.00)	0.77 (0.50-0.99)	0.93 (0.63-1.00)	0.93 (0.62-1.00)	0.78 (0.52-0.97)	0.92 (0.58-1.00)	0.77 (0.50-0.99)	0.93 (0.59-1.00)	0.93 (0.62-1.00)
Other	0.00 (0.00-0.14)	0.00 (0.00-0.15)	0.00 (0.00-0.12)	0.00 (0.00-0.12)	0.00 (0.00-0.13)	0.00 (0.00-0.13)	0.00 (0.00-0.14)	0.00 (0.00-0.13)	0.00 (0.00-0.13)	0.00 (0.00-0.14)	0.00 (0.00-0.12)	0.00 (0.00-0.14)	0.00 (0.00-0.13)	0.00 (0.00-0.13)
Cryptosporidium spp.														
Percent of total disease that is foodborne*	0.15 (0.00-0.44)	0.15 (0.00-0.47)	0.16 (0.01-0.44)	0.11 (0.01-0.38)	0.16 (0.01-0.44)	0.09 (0.00-0.41)	0.08 (0.00-0.36)	0.10 (0.00-0.39)	0.11 (0.00-0.39)	0.09 (0.00-0.40)	0.10 (0.00-0.37)	0.10 (0.00-0.42)	0.10 (0.00-0.40)	0.10 (0.00-0.45)
Percent of foodborne disease attributed to:														
Dairy	0.03 (0.00-0.41)	0.04 (0.00-0.46)	0.05 (0.00-0.29)	0.08 (0.00-0.35)	0.08 (0.00-0.36)	0.04 (0.00-0.49)	0.02 (0.00-0.46)	0.04 (0.00-0.40)	0.04 (0.00-0.44)	0.05 (0.00-0.49)	0.02 (0.00-0.38)	0.02 (0.00-0.40)	0.02 (0.00-0.33)	0.02 (0.00-0.44)
Fruits	0.31 (0.00-0.78)	0.29 (0.00-0.78)	0.31 (0.02-0.71)	0.27 (0.01-0.65)	0.24 (0.02-0.63)	0.24 (0.02-0.66)	0.23 (0.02-0.63)	0.26 (0.01-0.66)	0.25 (0.00-0.67)	0.23 (0.01-0.65)	0.25 (0.00-0.63)	0.26 (0.02-0.69)	0.25 (0.00-0.61)	0.24 (0.02-0.65)
Vegetables	0.56 (0.08-0.91)	0.56 (0.07-0.92)	0.61 (0.14-0.88)	0.62 (0.18-0.88)	0.65 (0.19-0.89)	0.60 (0.13-0.90)	0.62 (0.15-0.91)	0.61 (0.19-0.91)	0.60 (0.18-0.91)	0.61 (0.18-0.91)	0.64 (0.21-0.92)	0.63 (0.13-0.92)	0.64 (0.28-0.92)	0.64 (0.16-0.92)

Other	0.00 (0.00-0.21)	0.00 (0.00-0.21)	0.00 (0.00-0.09)	0.00 (0.00-0.10)	0.00 (0.00-0.13)	0.02 (0.00-0.21)	0.01 (0.00-0.21)	0.02 (0.00-0.13)	0.02 (0.00-0.13)	0.02 (0.00-0.14)	0.01 (0.00-0.25)	0.01 (0.00-0.20)	0.01 (0.00-0.23)	0.01 (0.00-0.16)

	AFR D	***AFR E***	***AMR A***	***AMR B***	***AMR D***	***EMR B***	***EMR D***	***EUR A***	***EUR B***	***EUR C***	***SEAR B***	***SEAR D***	***WPR A***	***WPR B***
Number of Experts	9	11	12	10	5	2	5	20	5	2	3	12	4	3
Echinococcus granulosus														
Percent of total disease that is foodborne*	0.21 (0.07-0.42)	0.20 (0.05-0.40)	0.20 (0.03-0.40)	0.20 (0.02-0.43)	0.21 (0.05-0.41)	0.21 (0.05-0.43)	0.21 (0.06-0.41)	0.21 (0.04-0.40)	0.21 (0.06-0.40)	0.21 (0.04-0.40)	0.21 (0.03-0.44)	0.20 (0.06-0.40)	0.20 (0.01-0.39)	0.21 (0.05-0.43)
Percent of foodborne disease attributed to:														
Fruits	0.22 (0.03-0.53)	0.22 (0.03-0.45)	0.22 (0.02-0.53)	0.23 (0.04-0.52)	0.22 (0.03-0.52)	0.21 (0.02-0.48)	0.23 (0.02-0.49)	0.22 (0.02-0.54)	0.21 (0.03-0.49)	0.23 (0.02-0.51)	0.22 (0.02-0.55)	0.21 (0.01-0.49)	0.21 (0.02-0.48)	0.21 (0.01-0.46)
Vegetables	0.77 (0.45-0.96)	0.77 (0.49-0.96)	0.77 (0.45-0.98)	0.76 (0.44-0.95)	0.77 (0.43-0.96)	0.78 (0.50-0.97)	0.77 (0.49-0.98)	0.77 (0.40-0.97)	0.78 (0.48-0.96)	0.76 (0.47-0.97)	0.77 (0.42-0.97)	0.78 (0.50-0.98)	0.78 (0.49-0.98)	0.78 (0.51-0.98)
Other	0.00 (0.00-0.14)	0.00 (0.00-0.25)	0.00 (0.00-0.13)	0.00 (0.00-0.22)	0.00 (0.00-0.24)	0.00 (0.00-0.13)	0.00 (0.00-0.14)	0.00 (0.00-0.31)	0.00 (0.00-0.15)	0.00 (0.00-0.13)	0.00 (0.00-0.16)	0.00 (0.00-0.14)	0.00 (0.00-0.13)	0.00 (0.00-0.17)
Echinococcus multilocularis														
Percent of total disease that is foodborne*			0.51 (0.13-0.79)			0.43 (0.09-0.73)	0.48 (0.00-0.77)	0.52 (0.15-0.79)	0.45 (0.12-0.72)	0.44 (0.12-0.72)		0.58 (0.00-0.88)	0.51 (0.09-0.81)	0.48 (0.00-0.78)
Fruits			0.46 (0.06-0.89)			0.44 (0.07-0.86)	0.41 (0.00-1.00)	0.46 (0.13-0.89)	0.45 (0.11-0.79)	0.43 (0.09-0.76)		0.41 (0.00-1.00)	0.45 (0.07-0.89)	0.41 (0.00-1.00)
Vegetables			0.54 (0.10-0.93)			0.54 (0.08-0.89)	0.59 (0.00-1.00)	0.53 (0.09-0.86)	0.55 (0.20-0.88)	0.54 (0.21-0.87)		0.59 (0.00-1.00)	0.55 (0.11-0.93)	0.58 (0.00-1.00)

Other			0.00 (0.00-0.01)			0.00 (0.00-0.10)	0.00 (0.00-0.00)	0.00 (0.00-0.06)	0.00 (0.00-0.09)	0.00 (0.00-0.11)		0.00 (0.00-0.00)	0.00 (0.00-0.01)	0.00 (0.00-0.22)
Entamoeba histolytica														
Percent of total disease that is foodborne*	0.30 (0.00-0.68)	0.30 (0.00-0.68)	0.25 (0.00-0.70)	0.21 (0.00-0.62)	0.17 (0.00-0.58)	0.24 (0.00-0.62)	0.28 (0.00-0.66)	0.33 (0.00-0.71)	0.30 (0.00-0.66)	0.26 (0.00-0.64)	0.26 (0.00-0.65)	0.25 (0.00-0.63)	0.25 (0.00-0.62)	0.27 (0.00-0.63)
Fruits	0.32 (0.05-0.66)	0.31 (0.05-0.66)	0.25 (0.05-0.59)	0.22 (0.05-0.57)	0.17 (0.01-0.57)	0.33 (0.04-0.67)	0.33 (0.04-0.68)	0.30 (0.04-0.61)	0.33 (0.04-0.67)	0.34 (0.05-0.67)	0.29 (0.05-0.62)	0.30 (0.04-0.61)	0.32 (0.05-0.64)	0.32 (0.04-0.64)
Vegetables	0.64 (0.29-0.90)	0.64 (0.29-0.90)	0.71 (0.38-0.91)	0.74 (0.39-0.92)	0.79 (0.39-0.96)	0.62 (0.28-0.90)	0.62 (0.28-0.89)	0.66 (0.35-0.90)	0.62 (0.27-0.90)	0.62 (0.28-0.89)	0.66 (0.34-0.90)	0.66 (0.35-0.90)	0.64 (0.32-0.90)	0.64 (0.32-0.89)
Other	0.03 (0.00-0.16)	0.03 (0.00-0.17)	0.00 (0.00-0.15)	0.00 (0.00-0.15)	0.00 (0.00-0.15)	0.03 (0.00-0.16)	0.03 (0.00-0.17)	0.02 (0.00-0.15)	0.03 (0.00-0.17)	0.03 (0.00-0.16)	0.03 (0.00-0.15)	0.03 (0.00-0.15)	0.03 (0.00-0.16)	0.03 (0.00-0.15)

*Percent of total disease estimated to be foodborne is taken from Hald et al. (2016) and reported here to provide perspective. Numbers in parentheses are 5th and 95th percentile values.
