# Supplementary material for: Attribution of global foodborne disease to specific foods: Findings from a World Health Organization structured expert elicitation
Source: PLoS One. 2017 Sep 14;12(9):e0183641. doi: 10.1371/journal.pone.0183641 (PMC5598938; doi:10.1371/journal.pone.0183641)
Supplement: S2 Table — Median proportion of total foodborne cases attributed to exposure to specified foods, by subregions for selected bacteria. (PDF) [file pone.0183641.s002.pdf]

S2 Table. Median proportion of total foodborne cases attributed to exposure to specified foods, by subregions for selected bacteria.

	AFR D	AFR E	AMR A	AMR B	AMR D	EMR B	EMR D	EUR A	EUR B	EUR C	SEAR B	SEAR D	WPR A	WPR B
Number of Experts	9	11	12	10	5	2	5	20	5	2	3	12	4	3
Brucella spp.														
Percent of total disease that is foodborne*	0.44 (0.10-0.68)	0.44 (0.06-0.70)	0.75 (0.28-0.93)	0.44 (0.09-0.69)	0.44 (0.09-0.72)	0.51 (0.08-0.80)	0.44 (0.07-0.70)	0.66 (0.23-0.90)	0.45 (0.09-0.71)	0.44 (0.10-0.73)	0.51 (0.07-0.81)	0.45 (0.07-0.70)	0.71 (0.28-0.92)	0.51 (0.07-0.80)
Percent of foodborne disease attributed to:														
Beef	0.05 (0.00-0.11)	0.05 (0.00-0.11)	0.03 (0.00-0.14)	0.05 (0.00-0.11)	0.05 (0.00-0.11)	0.03 (0.00-0.11)	0.05 (0.00-0.13)	0.03 (0.00-0.07)	0.05 (0.00-0.11)	0.05 (0.00-0.11)	0.05 (0.00-0.11)	0.05 (0.00-0.11)	0.03 (0.00-0.13)	0.05 (0.00-0.12)
Dairy	0.68 (0.50-0.86)	0.68 (0.45-0.87)	0.91 (0.62-0.96)	0.78 (0.60-0.90)	0.77 (0.58-0.89)	0.81 (0.66-0.93)	0.68 (0.49-0.86)	0.90 (0.66-0.96)	0.80 (0.61-0.91)	0.78 (0.58-0.89)	0.79 (0.57-0.90)	0.68 (0.49-0.87)	0.90 (0.65-0.96)	0.79 (0.50-0.90)
Goat, Lamb and other small ruminant meats	0.19 (0.01-0.37)	0.19 (0.00-0.44)	0.03 (0.00-0.28)	0.06 (0.00-0.23)	0.06 (0.00-0.26)	0.10 (0.00-0.22)	0.19 (0.00-0.38)	0.03 (0.00-0.26)	0.06 (0.00-0.26)	0.06 (0.00-0.25)	0.06 (0.00-0.27)	0.19 (0.00-0.38)	0.03 (0.00-0.24)	0.06 (0.00-0.33)
Other	0.05 (0.00-0.11)	0.05 (0.00-0.11)	0.01 (0.00-0.08)	0.02 (0.00-0.11)	0.05 (0.00-0.13)	0.01 (0.00-0.10)	0.05 (0.00-0.11)	0.01 (0.00-0.03)	0.02 (0.00-0.06)	0.05 (0.00-0.11)	0.02 (0.00-0.08)	0.05 (0.00-0.11)	0.01 (0.00-0.06)	0.02 (0.00-0.06)

*Percent of total disease estimated to be foodborne is taken from Hald et al. (2016) and reported here to provide perspective. Numbers in parentheses are 5th and 95th percentile values.

	AFR D	AFR E	AMR A	AMR B	AMR D	EMR B	EMR D	EUR A	EUR B	EUR C	SEAR B	SEAR D	WPR A	WPR B
Number of Experts	9	11	12	10	5	2	5	20	5	2	3	12	4	3
Campylobacter spp.														
Percent of total disease that is foodborne*	0.57 (0.31-0.77)	0.57 (0.29-0.77)	0.73 (0.38-0.91)	0.68 (0.41-0.82)	0.67 (0.37-0.81)	0.67 (0.38-0.82)	0.67 (0.41-0.82)	0.76 (0.44-0.93)	0.66 (0.34-0.87)	0.66 (0.34-0.87)	0.57 (0.27-0.81)	0.51 (0.03-0.79)	0.68 (0.40-0.89)	0.57 (0.25-0.82)
Percent of foodborne disease attributed to:														
Beef	0.06	0.06	0.15	0.06	0.06	0.05	0.06	0.16	0.05	0.05	0.08	0.05	0.17	0.09
	(0.00, 0.31)	(0.00, 0.34)	(0.00, 0.33)	(0.00, 0.32)	(0.00, 0.27)	(0.00, 0.25)	(0.00, 0.32)	(0.00, 0.37)	(0.00, 0.29)	(0.00, 0.29)	(0.00, 0.32)	(0.00, 0.28)	(0.00, 0.34)	(0.00, 0.30)
Dairy	0.13	0.14	0.07	0.12	0.13	0.10	0.13	0.07	0.15	0.14	0.15	0.13	0.04	0.13
	(0.00, 0.33)	(0.01, 0.32)	(0.00, 0.38)	(0.00, 0.30)	(0.00, 0.31)	(0.00, 0.25)	(0.01, 0.31)	(0.00, 0.27)	(0.00, 0.32)	(0.00, 0.31)	(0.00, 0.41)	(0.00, 0.40)	(0.00, 0.23)	(0.00, 0.30)
Fruits and nuts	0.00	0.00	0.00	0.00	0.00	0.00	0.00	0.01	0.01	0.01	0.03	0.03	0.00	0.03
	(0.00, 0.12)	(0.00, 0.12)	(0.00, 0.10)	(0.00, 0.10)	(0.00, 0.10)	(0.00, 0.08)	(0.00, 0.10)	(0.00, 0.07)	(0.00, 0.10)	(0.00, 0.11)	(0.00, 0.18)	(0.00, 0.17)	(0.00, 0.07)	(0.00, 0.16)
Goat, lamb and other small ruminant meats	0.10	0.10	0.04	0.10	0.09	0.08	0.11	0.04	0.07	0.07	0.14	0.11	0.05	0.07
	(0.00, 0.28)	(0.00, 0.29)	(0.00, 0.18)	(0.00, 0.26)	(0.00, 0.27)	(0.00, 0.26)	(0.00, 0.32)	(0.00, 0.17)	(0.00, 0.28)	(0.00, 0.28)	(0.00, 0.37)	(0.00, 0.33)	(0.00, 0.20)	(0.00, 0.29)
Pork	0.00	0.00	0.04	0.00	0.00	0.00	0.00	0.06	0.08	0.08	0.14	0.16	0.04	0.13
	(0.00, 0.17)	(0.00, 0.19)	(0.00, 0.21)	(0.00, 0.26)	(0.00, 0.25)	(0.00, 0.05)	(0.00, 0.08)	(0.00, 0.25)	(0.00, 0.24)	(0.00, 0.24)	(0.00, 0.38)	(0.00, 0.36)	(0.00, 0.24)	(0.00, 0.34)
Poultry meat	0.53	0.50	0.50	0.51	0.52	0.38	0.53	0.50	0.42	0.43	0.31	0.31	0.55	0.38
	(0.25, 0.75)	(0.21, 0.73)	(0.21, 0.75)	(0.23, 0.75)	(0.22, 0.74)	(0.16, 0.66)	(0.22, 0.75)	(0.22, 0.73)	(0.20, 0.66)	(0.20, 0.67)	(0.03, 0.60)	(0.05, 0.61)	(0.24, 0.77)	(0.15, 0.63)
Vegetables	0.06	0.06	0.05	0.06	0.06	0.33	0.06	0.03	0.09	0.09	0.06	0.05	0.04	0.05

	(0.00, 0.24)	(0.00, 0.24)	(0.00, 0.20)	(0.00, 0.23)	(0.00, 0.23)	(0.03, 0.58)	(0.00, 0.24)	(0.00, 0.21)	(0.00, 0.28)	(0.00, 0.29)	(0.00, 0.23)	(0.00, 0.39)	(0.00, 0.22)	(0.00, 0.28)
Other foods	0.00	0.00	0.00	0.00	0.00	0.00	0.00	0.02	0.01	0.01	0.00	0.00	0.00	0.00
	(0.00, 0.04)	(0.00, 0.04)	(0.00, 0.15)	(0.00, 0.04)	(0.00, 0.06)	(0.00, 0.02)	(0.00, 0.03)	(0.00, 0.15)	(0.00, 0.10)	(0.00, 0.09)	(0.00, 0.03)	(0.00, 0.07)	(0.00, 0.15)	(0.00, 0.03)

*Percent of total disease estimated to be foodborne is taken from Hald et al. (2016) and reported here to provide perspective.

	AFR D	AFR E	AMR A	AMR B	AMR D	EMR B	EMR D	EUR A	EUR B	EUR C	SEAR B	SEAR D	WPR A	WPR B
Number of Experts	9	11	12	10	5	2	5	20	5	2	3	12	4	3
Non-typhoidal *Salmonella* spp.														
Percent of total disease that is foodborne*	0.46 (0.13-0.74)	0.46 (0.10-0.73)	0.73 (0.38-0.91)	0.49 (0.09-0.74)	0.50 (0.14-0.75)	0.50 (0.18-0.75)	0.50 (0.19-0.74)	0.76 (0.47-0.94)	0.62 (0.31-0.84)	0.62 (0.32-0.84)	0.58 (0.23-0.84)	0.54 (0.00-0.85)	0.74 (0.45-0.93)	0.57 (0.25-0.82)
Percent of foodborne disease attributed to:														
Beef	0.06	0.06	0.07	0.06	0.06	0.05	0.05	0.04	0.01	0.01	0.02	0.01	0.09	0.04
	(0.00, 0.19)	(0.00, 0.19)	(0.00, 0.26)	(0.00, 0.28)	(0.00, 0.26)	(0.00, 0.18)	(0.00, 0.17)	(0.00, 0.26)	(0.00, 0.22)	(0.00, 0.23)	(0.00, 0.19)	(0.00, 0.18)	(0.00, 0.30)	(0.00, 0.18)
Dairy	0.05	0.05	0.05	0.05	0.05	0.06	0.06	0.02	0.02	0.02	0.05	0.06	0.04	0.04
	(0.00, 0.19)	(0.00, 0.19)	(0.00, 0.24)	(0.00, 0.17)	(0.00, 0.18)	(0.01, 0.19)	(0.00, 0.16)	(0.00, 0.19)	(0.00, 0.20)	(0.00, 0.20)	(0.00, 0.21)	(0.00, 0.29)	(0.00, 0.20)	(0.00, 0.19)
Eggs	0.21	0.21	0.22	0.19	0.20	0.22	0.22	0.24	0.26	0.26	0.09	0.09	0.22	0.10
	(0.01, 0.40)	(0.01, 0.41)	(0.01, 0.50)	(0.01, 0.39)	(0.01, 0.40)	(0.03, 0.41)	(0.01, 0.41)	(0.00, 0.52)	(0.01, 0.53)	(0.03, 0.53)	(0.00, 0.31)	(0.00, 0.32)	(0.00, 0.48)	(0.00, 0.38)
Fish	0.02	0.02	0.00	0.02	0.02	0.02	0.02	0.00	0.00	0.00	0.01	0.01	0.00	0.01
	(0.00, 0.09)	(0.00, 0.07)	(0.00, 0.04)	(0.00, 0.06)	(0.00, 0.10)	(0.00, 0.07)	(0.00, 0.07)	(0.00, 0.16)	(0.00, 0.06)	(0.00, 0.06)	(0.00, 0.09)	(0.00, 0.09)	(0.00, 0.07)	(0.00, 0.08)

Fruits and Nuts	0.04	0.04	0.03	0.07	0.05	0.04	0.04	0.01	0.02	0.02	0.03	0.03	0.02	0.03
	(0.00, 0.17)	(0.00, 0.17)	(0.00, 0.14)	(0.00, 0.19)	(0.00, 0.19)	(0.00, 0.16)	(0.00, 0.17)	(0.00, 0.11)	(0.00, 0.14)	(0.00, 0.13)	(0.00, 0.23)	(0.00, 0.20)	(0.00, 0.12)	(0.00, 0.18)
Goat, lamb, and other small ruminant meats	0.05	0.05	0.02	0.04	0.04	0.05	0.05	0.02	0.02	0.02	0.07	0.06	0.03	0.05
	(0.00, 0.29)	(0.00, 0.27)	(0.00, 0.11)	(0.00, 0.24)	(0.00, 0.24)	(0.00, 0.29)	(0.00, 0.26)	(0.00, 0.11)	(0.00, 0.28)	(0.00, 0.27)	(0.00, 0.39)	(0.00, 0.38)	(0.00, 0.14)	(0.00, 0.31)
Grains	0.01	0.01	0.00	0.01	0.01	0.01	0.01	0.00	0.01	0.01	0.02	0.02	0.00	0.02
	(0.00, 0.04)	(0.00, 0.05)	(0.00, 0.05)	(0.00, 0.05)	(0.00, 0.05)	(0.00, 0.07)	(0.00, 0.07)	(0.00, 0.06)	(0.00, 0.04)	(0.00, 0.04)	(0.00, 0.08)	(0.00, 0.18)	(0.00, 0.04)	(0.00, 0.06)
Pork	0.05	0.05	0.12	0.05	0.05	0.04	0.04	0.24	0.19	0.19	0.16	0.14	0.18	0.17
	(0.00, 0.20)	(0.00, 0.24)	(0.00, 0.39)	(0.00, 0.28)	(0.00, 0.26)	(0.00, 0.16)	(0.00, 0.15)	(0.01, 0.50)	(0.01, 0.39)	(0.01, 0.41)	(0.00, 0.34)	(0.00, 0.33)	(0.01, 0.42)	(0.00, 0.42)
Poultry meat	0.33	0.32	0.22	0.30	0.30	0.35	0.35	0.20	0.19	0.19	0.26	0.22	0.17	0.24
	(0.09, 0.52)	(0.07, 0.52)	(0.01, 0.46)	(0.09, 0.51)	(0.07, 0.51)	(0.10, 0.53)	(0.11, 0.54)	(0.01, 0.49)	(0.01, 0.42)	(0.00, 0.42)	(0.04, 0.48)	(0.01, 0.44)	(0.01, 0.43)	(0.01, 0.45)
Oils	0.00	0.00	0.00	0.00	0.00	0.00	0.00	0.00	0.01	0.01	0.02	0.02	0.00	0.02
	(0.00, 0.03)	(0.00, 0.03)	(0.00, 0.03)	(0.00, 0.03)	(0.00, 0.03)	(0.00, 0.04)	(0.00, 0.08)	(0.00, 0.03)	(0.00, 0.03)	(0.00, 0.04)	(0.00, 0.05)	(0.00, 0.05)	(0.00, 0.02)	(0.00, 0.05)
Seaweed	0.00	0.00	0.00	0.00	0.00	0.00	0.00	0.00	0.00	0.00	0.01	0.01	0.00	0.01
	(0.00, 0.05)	(0.00, 0.03)	(0.00, 0.03)	(0.00, 0.03)	(0.00, 0.04)	(0.00, 0.04)	(0.00, 0.04)	(0.00, 0.03)	(0.00, 0.04)	(0.00, 0.03)	(0.00, 0.05)	(0.00, 0.05)	(0.00, 0.03)	(0.00, 0.05)
Shellfish	0.01	0.01	0.01	0.01	0.01	0.01	0.01	0.01	0.01	0.01	0.01	0.02	0.01	0.01
	(0.00, 0.09)	(0.00, 0.06)	(0.00, 0.06)	(0.00, 0.08)	(0.00, 0.10)	(0.00, 0.07)	(0.00, 0.07)	(0.00, 0.06)	(0.00, 0.08)	(0.00, 0.08)	(0.00, 0.07)	(0.00, 0.11)	(0.00, 0.10)	(0.00, 0.08)
Vegetables	0.05	0.05	0.09	0.08	0.07	0.05	0.05	0.05	0.07	0.07	0.05	0.06	0.08	0.05

	(0.00, 0.20)	(0.00, 0.20)	(0.00, 0.25)	(0.00, 0.24)	(0.00, 0.25)	(0.00, 0.19)	(0.00, 0.19)	(0.00, 0.23)	(0.00, 0.28)	(0.00, 0.28)	(0.00, 0.25)	(0.00, 0.29)	(0.00, 0.23)	(0.00, 0.27)
Other foods	0.00	0.00	0.00	0.00	0.00	0.00	0.00	0.00	0.00	0.00	0.00	0.00	0.00	0.00
	(0.00, 0.08)	(0.00, 0.01)	(0.00, 0.08)	(0.00, 0.01)	(0.00, 0.01)	(0.00, 0.01)	(0.00, 0.01)	(0.00, 0.08)	(0.00, 0.03)	(0.00, 0.03)	(0.00, 0.01)	(0.00, 0.01)	(0.00, 0.08)	(0.00, 0.04)

*Percent of total disease estimated to be foodborne is taken from Hald et al. (2016) and reported here to provide perspective. Numbers in parentheses are 5th and 95th percentile values.

	AFR D	AFR E	AMR A	AMR B	AMR D	EMR B	EMR D	EUR A	EUR B	EUR C	SEAR B	SEAR D	WPR A	WPR B
Shiga toxin-producing *Escherichia coli* (STEC)														
Percent of total disease that is foodborne	0.42 (0.19-0.66)	0.43 (0.14-0.66)	0.59 (0.19-0.84)	0.53 (0.24-0.73)	0.53 (0.24-0.75)	0.53 (0.24-0.76)	0.52 (0.26-0.75)	0.60 (0.26-0.83)	0.49 (0.15-0.75)	0.49 (0.15-0.75)	0.41 (0.10-0.70)	0.40 (0.08-0.71)	0.57 (0.25-0.82)	0.43 (0.12-0.73)
Percent of foodborne disease attributed to:														
Beef	0.40 (0.09-0.65)	0.41 (0.09-0.66)	0.54 (0.29-0.77)	0.47 (0.20-0.69)	0.48 (0.20-0.69)	0.42 (0.11-0.66)	0.42 (0.13-0.67)	0.42 (0.17-0.67)	0.47 (0.18-0.68)	0.47 (0.18-0.68)	0.08 (0.00-0.43)	0.07 (0.00-0.41)	0.44 (0.19-0.70)	0.20 (0.01-0.51)
Dairy	0.14 (0.00-0.34)	0.14 (0.00-0.33)	0.13 (0.00-0.31)	0.13 (0.00-0.30)	0.13 (0.00-0.32)	0.14 (0.00-0.35)	0.14 (0.00-0.35)	0.17 (0.01-0.37)	0.17 (0.00-0.35)	0.17 (0.00-0.35)	0.14 (0.00-0.46)	0.14 (0.00-0.44)	0.14 (0.00-0.34)	0.14 (0.00-0.35)
Fruits	0.05 (0.00-0.24)	0.05 (0.00-0.22)	0.02 (0.00-0.20)	0.05 (0.00-0.21)	0.05 (0.00-0.21)	0.05 (0.00-0.22)	0.05 (0.00-0.22)	0.02 (0.00-0.18)	0.01 (0.00-0.21)	0.01 (0.00-0.21)	0.12 (0.00-0.38)	0.11 (0.00-0.33)	0.01 (0.00-0.13)	0.09 (0.00-0.33)
Goat, Lamb and other small ruminant meats	0.23 (0.01-0.48)	0.22 (0.02-0.44)	0.07 (0.00-0.24)	0.14 (0.00-0.35)	0.14 (0.00-0.35)	0.24 (0.00-0.49)	0.24 (0.00-0.49)	0.08 (0.00-0.29)	0.06 (0.00-0.30)	0.06 (0.00-0.32)	0.26 (0.01-0.59)	0.26 (0.01-0.59)	0.17 (0.00-0.36)	0.20 (0.00-0.46)
Vegetables	0.09 (0.00-0.29)	0.09 (0.00-0.29)	0.17 (0.00-0.41)	0.14 (0.01-0.33)	0.14 (0.00-0.34)	0.09 (0.00-0.32)	0.09 (0.00-0.31)	0.14 (0.01-0.37)	0.14 (0.00-0.38)	0.14 (0.00-0.39)	0.12 (0.00-0.40)	0.13 (0.00-0.45)	0.12 (0.00-0.42)	0.11 (0.00-0.44)
Other	0.00 (0.00-0.12)	0.00 (0.00-0.11)	0.00 (0.00-0.12)	0.00 (0.00-0.10)	0.00 (0.00-0.10)	0.00 (0.00-0.12)	0.00 (0.00-0.12)	0.00 (0.00-0.14)	0.00 (0.00-0.09)	0.00 (0.00-0.10)	0.00 (0.00-0.11)	0.00 (0.00-0.10)	0.00 (0.00-0.10)	0.00 (0.00-0.08)

*Percent of total disease estimated to be foodborne is taken from Hald et al. (2016) and reported here to provide perspective. Numbers in parentheses are 5th and 95th percentile values.

	AFR D	AFR E	AMR A	AMR B	AMR D	EMR B	EMR D	EUR A	EUR B	EUR C	SEAR B	SEAR D	WPR A	WPR B
Toxoplasma gondii														
Percent of total disease that is foodborne*	0.48 (0.24-0.76)	0.42 (0.20-0.70)	0.60 (0.30-0.81)	0.52 (0.27-0.77)	0.53 (0.27-0.77)	0.52 (0.27-0.80)	0.53 (0.29-0.77)	0.61 (0.35-0.82)	0.45 (0.23-0.76)	0.53 (0.31-0.78)	0.52 (0.26-0.77)	0.43 (0.09-0.73)	0.60 (0.33-0.81)	0.53 (0.29-0.77)
Percent of foodborne disease attributed to:														
Beef	0.31 (0.03-0.61)	0.33 (0.07-0.60)	0.34 (0.08-0.58)	0.27 (0.01-0.55)	0.33 (0.00-0.60)	0.20 (0.03-0.39)	0.18 (0.03-0.37)	0.25 (0.00-0.51)	0.38 (0.08-0.62)	0.25 (0.00-0.54)	0.23 (0.06-0.45)	0.19 (0.04-0.37)	0.30 (0.09-0.55)	0.26 (0.06-0.47)
Dairy	0.00 (0.00-0.31)	0.00 (0.00-0.16)	0.00 (0.00-0.13)	0.00 (0.00-0.14)	0.00 (0.00-0.13)	0.00 (0.00-0.12)	0.00 (0.00-0.18)	0.00 (0.00-0.12)	0.00 (0.00-0.13)	0.00 (0.00-0.13)	0.00 (0.00-0.13)	0.00 (0.00-0.12)	0.00 (0.00-0.14)	0.00 (0.00-0.11)
Eggs	0.00 (0.00-0.03)	0.00 (0.00-0.02)	0.00 (0.00-0.02)	0.00 (0.00-0.02)	0.00 (0.00-0.02)	0.00 (0.00-0.03)	0.00 (0.00-0.03)	0.00 (0.00-0.02)	0.00 (0.00-0.02)	0.00 (0.00-0.02)	0.00 (0.00-0.02)	0.00 (0.00-0.03)	0.00 (0.00-0.03)	0.00 (0.00-0.02)
Fruits	0.03 (0.00-0.18)	0.03 (0.00-0.16)	0.02 (0.00-0.17)	0.02 (0.00-0.14)	0.02 (0.00-0.15)	0.03 (0.00-0.16)	0.03 (0.00-0.17)	0.07 (0.00-0.22)	0.04 (0.00-0.13)	0.04 (0.00-0.14)	0.04 (0.00-0.19)	0.03 (0.00-0.19)	0.03 (0.00-0.14)	0.03 (0.00-0.17)
Goat, Lamb and other small ruminant meats	0.28 (0.05-0.61)	0.18 (0.02-0.45)	0.12 (0.00-0.33)	0.10 (0.00-0.41)	0.08 (0.00-0.38)	0.42 (0.12-0.65)	0.44 (0.14-0.69)	0.11 (0.00-0.42)	0.09 (0.01-0.37)	0.18 (0.03-0.48)	0.19 (0.02-0.46)	0.16 (0.01-0.47)	0.14 (0.00-0.43)	0.18 (0.01-0.47)
Pork	0.06 (0.00-0.34)	0.13 (0.00-0.41)	0.16 (0.00-0.50)	0.22 (0.01-0.54)	0.22 (0.02-0.52)	0.02 (0.00-0.30)	0.02 (0.00-0.31)	0.20 (0.02-0.51)	0.12 (0.00-0.49)	0.12 (0.00-0.50)	0.17 (0.00-0.41)	0.15 (0.00-0.44)	0.12 (0.00-0.49)	0.14 (0.00-0.41)
Poultry meat	0.05 (0.00-0.32)	0.06 (0.00-0.34)	0.08 (0.00-0.34)	0.07 (0.00-0.34)	0.07 (0.00-0.34)	0.07 (0.00-0.33)	0.00 (0.00-0.34)	0.09 (0.00-0.35)	0.07 (0.00-0.35)	0.07 (0.00-0.32)	0.09 (0.00-0.36)	0.14 (0.00-0.38)	0.11 (0.00-0.40)	0.14 (0.00-0.39)
Vegetables	0.14 (0.00-0.39)	0.16 (0.00-0.37)	0.15 (0.00-0.44)	0.19 (0.00-0.46)	0.17 (0.00-0.45)	0.21 (0.00-0.41)	0.23 (0.00-0.47)	0.17 (0.00-0.41)	0.18 (0.00-0.40)	0.21 (0.01-0.47)	0.21 (0.00-0.42)	0.22 (0.00-0.46)	0.15 (0.00-0.39)	0.15 (0.00-0.36)

Other	0.00 (0.00-0.14)	0.00 (0.00-0.12)	0.00 (0.00-0.13)	0.00 (0.00-0.14)	0.00 (0.00-0.12)	0.00 (0.00-0.14)	0.00 (0.00-0.14)	0.00 (0.00-0.13)	0.00 (0.00-0.14)	0.00 (0.00-0.12)	0.00 (0.00-0.13)	0.02 (0.00-0.14)	0.00 (0.00-0.12)	0.00 (0.00-0.12)

*Percent of total disease estimated to be foodborne is taken from Hald et al. (2016) and reported here to provide perspective. Numbers in parentheses are 5th and 95th percentile values.
