# Supplementary material for: Attribution of global foodborne disease to specific foods: Findings from a World Health Organization structured expert elicitation
Source: PLoS One. 2017 Sep 14;12(9):e0183641. doi: 10.1371/journal.pone.0183641 (PMC5598938; doi:10.1371/journal.pone.0183641)
Supplement: S3 File — Percent of foodborne incidence attributable to food-specific exposures (means and medians). (PDF) [file pone.0183641.s005.pdf]

Supplementary Online Material for

Estimates of the Relative Contributions to the Burden of Disease due to Selected Foodborne Hazards: a World Health Organization Expert Elicitation

Roger M. Cooke and Willy Aspinall

January 14, 2015

Performance Measures and Combination: The classical model

1. Calibration
2. Information
3. Combination: Decision maker

Applications of the Classical Model by Subject Area

1. Nuclear: Reports published as a result of the joint EC/USNRC Project on uncertainty analysis of probabilistic accident consequence codes (under the Third EC-Framework Programme)
2. Reports published on the project uncertainty analysis of the probabilistic accident consequence code COSYMA using expert judgement (under the Fourth EC-Framework Programme)
3. Ecosystems / public health
4. Civil aviation / structural reliability
5. Information security
6. Natural hazards
7. Climate
8. Banking/Finance
9. Completed, publication in preparation

Performance Measures and Combination: The Classical Model

There are two generic, quantitative measures of expert performance, *calibration* and *information*. Loosely, calibration measures the statistical likelihood that a set of experimental results correspond, in a statistical sense, with an expert's assessments. More precisely, it is the p-value at which we would falsely reject the hypothesis that an expert's probability statements were accurate. In this study the 5, 50 and 95 percentiles, or quantiles, were elicited from each expert for each of the continuous variables. Hence, each expert divides the range of possible outcomes of each variable into 4 intervals: less than or equal to the 5% value, greater than the 5% value and less than or equal to the 50% value, etc. The probabilities for these intervals are expressed as a vector

$$p = (p_1, p_2, p_3, p_4) = (0.05, 0.45, 0.45, 0.05).$$

Calibration

If N quantities are assessed, each expert may be regarded as a statistical hypothesis, namely that each realization falls in one of the four inter-quantile intervals with probability vector p. Suppose we have realizations $x_1, \dots x_N$ of these quantities. We may then form the sample distribution of the expert's inter quantile intervals as:

$s_1(e) = \#\{ i \mid x_i \leq 5\%\ quantile\} / N$
$s_2(e) = \#\{ i \mid 5\%\ quantile < x_i \leq 50\%\ quantile\} / N$
$s_3(e) = \#\{ i \mid 50\%\ quantile < x_i \leq 95\%\ quantile\} / N$
$s_4(e) = \#\{ i \mid 95\%\ quantile < x_i \} / N$
$s(e) = (s_1, \ldots s_4)$

Note that the sample distribution depends on the expert *e*. If the realizations are indeed drawn independently from a distribution with quantiles as stated by the expert then the quantity

$$2NI(s(e) \mid p) = 2N \sum_{i=1..4} s_i \ln(s_i / p_i) \qquad (1)$$

is asymptotically distributed as a chi-square variable with 3 degrees of freedom. This is the likelihood ratio statistic, and I(s | p) is the relative information of distribution *s* with respect to *p*. Extracting the leading term of the logarithm yields the familiar chi-square test statistic for goodness of fit. There are advantages in using the form in (1) (Cooke 1991).

If after a few realizations the expert were to see that all realization fell outside his 90% central confidence intervals, they might conclude that these intervals were too narrow and could seek to broaden them on subsequent assessments. This means that for this expert the uncertainty distributions are *not* independent - they learn from the realizations. Expert learning is not a goal of an expert judgment study. Rather, the problem owner wants experts who do not need to learn from the elicitation. Independence is not an assumption about the expert's distribution but a desideratum of the problem owner. Hence the decision maker (see below) scores expert *e* as the statistical likelihood of the hypothesis

H_e: *"the inter quantile interval containing the true value for each variable is drawn independently from probability vector p."*

A simple test for this hypothesis uses the test statistic (1), and the likelihood, or *p*-value, or **calibration score** of this hypothesis, is:

$$Cal(e) = p\text{-}value(e) = Prob\{2NI(s(e)\ p) \geq r \mid H_e\}$$

where *r* is the value of (1) based on the observed values $x_1, \ldots x_N$. It is the probability under hypothesis H_e that a deviation at least as great as *r* should be observed on *N* realizations if H_e were true. Calibration scores are absolute and can be compared across studies. However it is appropriate to equalize the power of the different hypothesis tests by equalizing the effective number of realizations. To compare scores on two data sets with *N* and *N' realizations*, we simply use the minimum of *N* and *N'* in (1), without changing the sample distribution *s*.

Although the calibration score uses the language of simple hypothesis testing, it must be emphasized that we are not rejecting expert-hypotheses; rather we are using this language to measure the degree to which the data supports the hypothesis that the expert's probabilities are accurate. Low scores, near zero, mean that it is unlikely that the expert’s probabilities are correct. High scores, near 1, indicate good support.

Information
The second scoring variable is information. Loosely, the information in a distribution is the degree to which the distribution is concentrated. Information cannot be measured absolutely, but only with respect to a background measure. Being concentrated or 'spread out' is measured relative to some other distribution.

Measuring information requires associating a density with each assessment of each expert. To do this, we use the unique density that complies with the experts' quantiles and is minimally informative with respect to the background measure. This density can easily be found with the method of Lagrange multipliers. For a uniform background measure, the density is constant between the assessed quantiles. The background measure is not elicited from experts as indeed it must be the same for all experts; instead it is chosen by the analyst.

The uniform and log-uniform background measures require an *intrinsic range* on which these measures are concentrated. The classical model implements the so-called k% overshoot rule: for each item we consider the smallest interval $I = [L, U]$ containing all the assessed quantiles of all experts and the realization, if known. This interval is extended to

$$I^* = [L^*, U^*]; L^* = L - k(U\text{-}L)/100; \;\; U^* = U + k(U\text{-}L)/100.$$

The value of k is chosen by the analyst. A large value of k tends to make all experts look quite informative, and tends to suppress the relative differences in information scores. The **information score** of expert e on assessments for uncertain quantities $1\ldots N$ is

$$Inf\,(e) = \text{Average Relative information w.r.t. Background} = (1/N) \textstyle\sum_{i = 1..N} I(f_{e,i} \mid g_i)$$

where g_i is the background density for variable i and $f_{e,i}$ is expert e's density for item i. This is proportional to the relative information of the expert's joint distribution given the background, under the assumption that the variables are independent. As with calibration, the assumption of independence here reflects a desideratum of the decision maker and not an elicited feature of the expert's joint distribution. The information score does not depend on the realizations. An expert can seek to achieve a high information score by choosing quantiles that are very close together. But the information score of expert e depends on the group intrinsic range, which depends arising on the assessments of the other experts involved. Hence, information scores cannot be compared across studies.

The above information score is chosen because it is familiar, tail insensitive, scale invariant and 'slow'. The latter property means that relative information is a slow function; large changes in the expert assessments produce only modest changes in the information score. This contrasts with the likelihood function in the calibration score, which is a very 'fast' function. This causes the product of calibration and information to be driven by the calibration score.

Combination: Decision Maker
The **combined score** of expert e will serve as an (unnormalized) weight for e:

$$w_\alpha(e) = Cal\,(e) \times Inf\,(e) \times \mathbb{1}_\alpha(Cal(e) \geq \alpha), \qquad (2)$$

where $\mathbb{1}_{\alpha}(Cal(e)\alpha) = 1$ if $Cal(e) \geq \alpha$, and is zero otherwise. The combined score thus depends on α; if $Cal(e)$ falls below cut-off level α, expert *e* is unweighted. The presence of a cut-off level is imposed by the requirement that the combined score be an asymptotically strictly proper scoring rule. That is, each expert maximizes their long run expected score by and only by ensuring that their probabilities $p= (0.05, 0.45, 0.45, 0.05)$ correspond to their true beliefs (Cooke, 1991). α is similar to a significance level in simple hypothesis testing, but its purpose is to measure 'goodness' and not to reject hypotheses.

A combination of expert assessments is called a 'decision maker' (DM). All decision makers discussed here are examples of linear pooling; the classical model is essentially a method for deriving weights in a linear pool. 'Good expertise' corresponds to good calibration (high statistical likelihood, high p-value) and high information. Weights that reward good expertise and pass these virtues on to the decision maker are desired.

The reward aspect of weights is very important. We could simply solve the following optimization problem: find a set of weights such that the linear pool under these weights maximizes the product of calibration and information. Solving this problem on real data, one finds that the weights do not generally reflect the performance of the individual experts. As an expert's influence on the decision maker should not appear haphazard, and 'gaming' the system with assessments tilted to achieve a desired outcome should be discouraged, we must impose a strictly scoring rule constraint on the weighting scheme.

The scoring rule constraint requires the term $_{\alpha}(Cal(e) \geq \alpha)$ in eq (2), but does not indicate what value of α we should select. Therefore, we choose α to maximize the combined score of the resulting decision maker. Let $DM_{\alpha}(i)$ be the result of linear pooling for any item *i* with weights proportional to (2):

$$DM_{\alpha}(i) = \sum_{e=1,..E} w_{\alpha}(e) f_{e,i} \; / \sum_{e=1,..E} w_{\alpha}(e) \tag{3}$$

The *optimized global weight DM* is $DM_{\alpha*}$ where α* maximizes

$$calibration\ score(DM_{\alpha*}) \times information\ score(DM_{\alpha*}). \tag{4}$$

This weight is termed global as the information score is based on all the assessed calibration items.

A variation on this scheme allows a different set of weights to be used for each item. This is accomplished by using information scores for each item rather than the average information score:

$$w_{\alpha}(e,i) = 1_{\alpha}(calibration\ score) \times calibration\ score(e) \times I(f_{e,i} \mid g_i) \tag{5}$$

For each α we define the *Item weight* DM_{α} for item *i* as

$$IDM_{\alpha}(i) = \sum_{e=1,..E} w_{\alpha}(e,i) f_{e,i} \; / \sum_{e=1,..E} w_{\alpha}(e,i) \tag{6}$$

The *optimized item weight DM* is $IDM_{\alpha*}$ where α* maximizes

$$calibration\ score(IDM_{\alpha}) \times information\ score(IDM_{\alpha}). \quad (7)$$

The non-optimized versions of the global and item weight DM's are obtained by setting $\alpha = 0$.

Item weights are potentially more attractive as they allow an expert to up- or down- weight judgments for individual items according to how much they feels they know about that particular item. 'Knowing less' means choosing quantiles farther apart and lowering the information score for that item. Of course, good performance of item weights requires that experts successfully perform this up-down weighting. Anecdotal evidence suggests that item weights improve over global weights as the experts receive more training in probabilistic assessment. Both item weights and global weights can be described as optimal weights under a strictly proper scoring rule constraint. With both global and item weights, calibration strongly dominates over information, and information serves to modulate between more or less equally well calibrated experts.

Since any combination of expert distributions yields assessments for the calibration variables, any combination can be evaluated on the calibration variables. In particular, we can compute the calibration and the information of any proposed decision maker. We should hope that the decision maker would perform better than the result of simple averaging, called the *equal weight DM* (EW), and we should also hope that the proposed DM is not worse than the best expert in the panel. The global and item weight DM's discussed above (optimized or not) are *Performance weighted DM's* (PW). In general the optimized global weight DM is used, unless the optimized item weight DM is markedly superior.

The expert judgment processing software EXCALIBUR may be freely downloaded at http://www.lighttwist.net/wp/excalibur

Applications of the Classical Model by Subject Area

1. *Nuclear: Reports published as a result of the Joint EC/USNRC Project on uncertainty analysis of probabilistic accident consequence codes (under the Third EC-Framework Programme)*

1.1. F.T. Harper, L.H.J. Goossens, R.M. Cooke, S.C. Hora, M.L. Young, J. Päsler-Sauer, L.A. Miller, B. Kraan, C. Lui, M.D. McKay, J.C. Helton and J.A. Jones Probabilistic accident consequence uncertainty study: Dispersion and deposition uncertainty assessment (1994) Prepared for U.S. Nuclear Regulatory Commission and Commission of European Communities NUREG/CR-6244, EUR 15855 EN, SAND94-1453, Washington/USA, and Brussels-Luxembourg, November 1994, published January 1995. Volume I: Main report, Volume II: Appendices A and B, Volume III: Appendices C, D, E, F, G, H

1.2. R.M. Cooke, L.H.J. Goossens and B.C.P. Kraan *Methods for CEC\USNRC accident consequence uncertainty analysis of dispersion and deposition - Performance based aggregating of expert judgements and PARFUM method for capturing modeling uncertainty* (1995) Prepared for the Commission of European Communities, EUR 15856, Brussels-Luxembourg, June 1994, published 1995

1.3. J. Brown, L.H.J. Goossens, F.T. Harper, B.C.P. Kraan, F.E. Haskin, M.L. Abbott, R.M. Cooke, M.L. Young, J.A. Jones S.C. Hora, A. Rood and J. Randall (1997) *Probabilistic accident consequence uncertainty study: Food chain uncertainty assessment* Prepared for U.S. Nuclear Regulatory Commission and Commission of European Communities NUREG/CR-6523, EUR 16771, SAND97-0335 Washington/USA, and Brussels-Luxembourg, March 1997, published June 1997. Volume 1: Main report, Volume 2: Appendices.

1.4. L.H.J. Goossens, J. Boardman, F.T. Harper, B.C.P. Kraan, R.M. Cooke, M.L. Young, J.A. Jones and S.C. Hora Probabilistic accident consequence uncertainty study: *Uncertainty assessment for deposited material and external doses* (1997) Prepared for U.S. Nuclear Regulatory Commission and Commission of European Communities, NUREG/CR-6526, EUR 16772, SAND97-2323 Washington/USA, and Brussels-Luxembourg, September 1997, published December 1997. Volume 1: Main report, Volume 2: Appendices

1.5. F.E. Haskin, F.T. Harper, L.H.J. Goossens, B.C.P. Kraan, J.B. Grupa and J. Randall (1997) *Probabilistic accident consequence uncertainty study: Early health effects uncertainty assessment* Prepared for U.S. Nuclear Regulatory Commission and Commission of European Communities NUREG/CR-6545, EUR 16775, SAND97-2689 Washington/USA, and Brussels-Luxembourg, November 1997, published December 1997. Volume 1: Main report, Volume 2: Appendices

1.6. M. Little, C.M. Muirhead, L.H.J. Goossens, F.T. Harper, B.C.P. Kraan, R.M. Cooke and S.C. Hora (1997) *Probabilistic accident consequence uncertainty study: Late health effects uncertainty assessment* Prepared for U.S. Nuclear Regulatory Commission and Commission of European Communities, NUREG/CR-6555, EUR 16774, SAND97-2322 Washington/USA, and Brussels-Luxembourg, September 1997, published December 1997. Volume 1: Main report, Volume 2: Appendices

1.7. L.H.J. Goossens, J.D. Harrison, F.T. Harper, B.C.P. Kraan, R.M. Cooke and S.C. Hora (1998) *Probabilistic accident consequence uncertainty study: Uncertainty assessment for internal dosimetry* Prepared for U.S. Nuclear Regulatory Commission and Commission of European Communities, NUREG/CR-6571, EUR 16773, SAND98-0119 Washington/USA, and Brussels-Luxembourg, February 1998, published April 1998 Volume 1: Main report, Volume 2: Appendices

2. *Reports published on the project uncertainty analysis of the probabilistic accident consequence code COSYMA using expert judgement (under the Fourth EC-Framework Programme)*

2.1. *Radiation Protection and Dosimetry Special Issue*, vol. 90 no 3, 2000.

2.2. R.M. Cooke, L.H.J. Goossens, B.C.P. Kraan (2000) Probabilistic Accident Consequence Uncertainty Assessment *Procedures Guide Using Expert Judgement* EUR 18820EN European Commission. Luxembourg 2000, Euratom.
2.3. L.H.J. Goossens, J.A. Jones, J. Ehrhardt, B.C.P. Kraan (2001) *Probabilistic Accident Consequence Uncertainty Assessment Countermeasures Uncertainty Assessment* EUR 18821EN European Commission. Luxembourg 2001, Euratom.
2.4. J.A. Jones, J. Ehrhardt, F. Fischer, I. Hasemann, L.H.J. Goossens, B.C.P. Kraan, R.M. Cooke (2001) *Probabilistic Accident Consequence Uncertainty Assessment Using COSYMA Uncertainty from the Atmospheric Dispersion and Deposition Module* EUR 18822EN European Commission. Luxembourg 2001, Euratom.
2.5. J.A. Jones, J. Brown, F. Fischer, I. Hasemann, L.H.J. Goossens, B.C.P. Kraan, R.M. Cooke (2001) *Probabilistic Accident Consequence Uncertainty Assessment Using COSYMA: Uncertainty from the Food Chain Module* EUR 18823EN European Commission. Luxembourg 2001, Euratom.
2.6. J.A. Jones, F. Fischer, I. Hasemann, L.H.J. Goossens, B.C.P. Kraan, R.M. Cooke (2001) *Probabilistic Accident Consequence Uncertainty Assessment Using COSYMA Uncertainty from the Health Effects Module* EUR 18824EN European Commission. Luxembourg 2001, Euratom.
2.7. J.A. Jones, F. Fischer, I. Hasemann, L.H.J. Goossens, B.C.P. Kraan, R.M. Cooke (2001) *Probabilistic Accident Consequence Uncertainty Assessment Using COSYMA Uncertainty from the Dose Module* EUR 18825EN European Commission. Luxembourg 2001, Euratom.
2.8. J.A. Jones, J. Ehrhardt, L.H.J. Goossens, F. Fischer, I. Hasemann, B.C.P. Kraan, R.M. Cooke (2001) *Probabilistic Accident Consequence Uncertainty Assessment Using COSYMA Uncertainty from the Complete System* EUR 18826EN European Commission. Luxembourg 2001, Euratom
2.9. J.A. Jones, B.C.P. Kraan, R.M. Cooke, L.H.J. Goossens, F. Fischer, I. Hasemann (2001) *Probabilistic Accident Consequence Uncertainty Assessment Using COSYMA Methodology and Processing Techniques* EUR 18827EN European Commission. Luxembourg 2001 Euratom.
2.10. Klügel, J-U., (2008) "Seismic Hazard Analysis — Quo vadis?" Earth-Science Reviews 88, 1–32

3. *Ecosystems / public health*

3.1. Tuomisto J.T, Wilson A., Cooke R.M., Tainio M., Evans J.S. (2005) Mortality in Kuwait due to PM from oil fires after the Gulf War: Combining expert elicitation assessments. *Epidemiology,* Volume 16 (5) September 2005 p. S74-S75
3.2. Evans J.S., Wilson A, Tuomisto JT, Tainio M, Cooke RM (2005) What risk assessment can tell us about the mortality impacts of the Kuwaiti oil fires. *Epidemiology,* Volume 16 (5) September 2005 p.S137-S138
3.3. Burgman, M. (2005) *Risk and Decisions for Conservation and Environmental Management*, Cambridge University Press, Cambridge, UK.
3.4. Van der Fels-Klerx, H.J., Cooke, R.M., Nauta, M.J., Goossens, L.H.J., Havelaar, A.H. (2005) A Structured Expert Judgement Study For A Model of Campylobacter Transmission During Broiler Chicken Processing. *Risk Analysis* 25 No. 1, 2005, pp 109-124.
3.5. Cooke, R.M. Wilson,A.M., Tuomisto,J.T. Morales,O. Tainio,M. and Evans, J.S.. (2007) A Probabilistic Characterization of the Relationship Between Fine Particulate Matter and Mortality: Elicitation of European Experts. *Environmental Science and Technology* 2007 Sep 15;41(18): pp 6598-6605.
3.6. Kurowicka, D., Cooke, R.M. Goossens, L. and Ale, B.J.M, (2008) Expert Judgment study for Placement Ladder Bowtie, *Safety Science,* Volume 46, Issue 6, July 2008, Pages 921–93.
3.7. Radboud J. Duintjer Tebbens, Kimberly M. Thompson, M.G. Myriam Hunink, M.D., Thomas M Mazzuchi, Daniel Lewandowski, Dorota Kurowicka, Roger M. Cooke, (2008) Uncertainty And Sensitivity Analyses Of A Dynamic Economic Evaluation Model For Vaccination Programs. *Medical Decision Making* 2008
3.8. Arie H. Havelaar, Ángela Vargas Galindo, Dorota Kurowicka, Roger M. Cooke (2008) Attribution of Foodborne Pathogens Using Structured Expert Elicitation, *Foodborne Pathogens and Disease*, October 2008, 5(5): 649-659. doi:10.1089/fpd.2008.0115

3.9. Rothlisberger, J.D., Lodge, D.M. Cooke, R.M. and Finnoff, D.C. (2009) Future declines of the binational Laurentian Great Lakes fisheries: recognizing the importance of environmental and cultural change. *Frontiers in Ecology and the Environment;* doi:10.1890/090002

3.10. Neslo R.E.J. and Cooke, R.M. (2011) Modeling and Validating Stakeholder Preferences with Probabilistic Inversion. *Applied Stochastic Models in Business and Industry.*

3.11. Kurowicka, D. Bucura, C. Cooke, R.M. and Havelaar, A. (2010) Probabilistic Inversion in Priority Setting of Emerging Zoonoses, *Risk Analysis,* Vol. 30, No. 5, 2010 DOI: 10.1111/j.1539-6924.2010.01378.x

3.12. Kurowicka, D. Nauta, M. Jozwiak,, K. 1 and Cooke, R.M. (2010) Updating Parameters of the Chicken Processing Line Model. *Risk Analys*is, Vol. 30, No. 6, 2010 DOI: 10.1111/j.1539-6924.2010.01379.x

3.13. Sarah J. Teck, Benjamin S. Halpern, Carrie V. Kappel, Fiorenza Micheli, Kimberly A. Selkoe, Caitlin M. Crain, Rebecca Martone, Christine Shearer, Joe Arvai, Baruch Fischhoff, Grant Murray, Rabin Neslo, and Roger Cooke (2010) Using expert judgment to estimate marine ecosystem vulnerability in the California Current. *Ecological Applications,* 20(5), 2010, pp. 1402–1416 2010 by the Ecological Society of America

3.14. Burgman, M. Carr, A. Godden, L. Gregory, R. McBride, M. Flander, L.Maguire, L..(2010) Redefining expertise and improving ecological judgement. Under review *Conservation Letters*.

3.15. Burgman,M. McBride,M., Ashton,R.,Speirs-Bridge,A., Flander, L., Wintle,B., Fidler, F., Rumpff, L.and Twardy,C. (2011) Perception of expertise and performance of experts: How do they compare?

3.16. Villie Flari, Qasim Chaudhry, Rabin Neslo and Roger Cooke (2011) Expert judgment based multi-criteria decision model to address uncertainties in risk assessment of nanotechnology-enabled food products *J Nanopart Res 13: 1813–1831; DOI 10.1007/s11051-011-0335-x*

3.17. Martin, T.G. Burgman, M.A., Fidler, F.F. , Kuhnert, P.M. , Low-Choy, S., Mcbride, M. , and Mengersen, K. (2011) Eliciting expert knowledge in conservation science *Conservation biology*, volume 26, no. 1, 29–38 2011 *Conservation biology* c _ 2011 Society for Conservation Biology Doi: 10.1111/j.1523-1739.2011.01806.x

3.18. Tyshenko, M.G., S. ElSaadany, T. Oraby, M. Laderoute, J. Wu, W. Aspinall and D. Krewski (2011) Risk Assessment and Management of Emerging Blood-Borne Pathogens in Canada: Xenotropic Murine Leukaemia Virus-Related Virus as a Case Study for the Use of a Precautionary Approach. Chapter in: *Risk Assessment* (ISBN 979-953-307-765-8).

3.19. Tyshenko, M.G., ElSaadany, S., Oraby, T., Darshan, S., Catford, A., Aspinall, W., Cooke, R. and Krewski, D. (2012) Expert judgement and re-elicitation for prion disease risk uncertainties. *International Journal of Risk Assessment and Management*, 16(1-3), 48-77. doi:10.1504/IJRAM.2012.047552

3.20. Tyshenko, M.G., S. ElSaadany, T. Oraby, S. Darshan, W. Aspinall, R. Cooke, A. Catford and D. Krewski (2011) Expert elicitation for the judgment of prion disease risk uncertainties. *J Toxicol Environ Health* A.; 74(2-4):261-285.

3.21. Oraby,T., Tyshenko, M.G., Westphal, M., Darshan, S., Croteau, M., Aspinall, W., Elsaadany, S., Cashman, N. and Krewski, D. (2011) Using Expert Judgments to Improve Chronic Wasting Disease Risk Management in Canada. *Journal of Toxicology and Environmental Health*, in press. Volume 74, Issue 2-4, 2011 Special Issue: Prion Research in Perspective 2010

3.22. Rothlisberger,J.D. Finnoff, D.C. Cooke,R.M. and Lodge, D.M. (2012) Ship-borne nonindigenous species diminish Great Lakes ecosystem services. *Ecosystems* (2012) 15: 462–476 DOI: 10.1007/s10021-012-9522-6

3.23. Halpern,B.S., Longo, C McLeod. C.L., Cooke, Roger M.., Fischhoff, B., Samhouri, J.F., Scarborough, C. (2013) Elicited preferences for components of ocean health in the California Current. *Marine Policy* 42 (2013) 68–73.

3.24. Cashman, N.R., Cheung, R., Aspinall, W., Wong, M. and Krewski, D. (2014) Expert Elicitation for the Judgment of Prion Disease Risk Uncertainties associated with Urine-derived and Recombinant Fertility Drugs. Submitted to: *Journal of Toxicology and Environmental Health*.

3.25. Cooke, R.M., Wittmann, M.E., Lodge, D.M., Rothlisberger, J.D., Rutherford E.S., Zhang, H. and Mason, D.M. (2014) Out-of-Sample Validation for Structured Expert Judgment of Asian Carp Establishment in Lake Erie. *Integrated Environmental Assessment and Management,* open access. DOI: 10.1002/ieam.1559 http://onlinelibrary.wiley.com/doi/10.1002/ieam.1559/abstract

3.26. Wittmann, M.E., Cooke, R.M., Rothlisberger, J.D., Rutherford, E. S., Zhang, H., Mason, D., Lodge, D.M. (2014b) Structured expert judgment to forecast species invasions: Bighead and silver carp in Lake Erie. *Conservation Biology* . DOI: 10.1111/cobi.12369 http://onlinelibrary.wiley.com/doi/10.1111/cobi.12369/full
3.27. Whitmann, M.E., Cooke, Roger M., Rothlisberger, J.D., Lodge, D.M. (2014) Using Structured Expert Judgment to Assess Invasive Species Prevention: Asian Carp and the Mississippi Great Lakes Hydrologic Connection. *Environmental Science and Technology*, accepted Jan 17, 2014, dx.doi.org/10.1021/es4043098 | Environ. Sci. Technol.

4. *Civil Aviation / structural reliability*

4.1. Cooke, R.M. & Jager, E. (1998) Failure Frequency of Underground Gas Pipelines. *Risk Analysis*, vol. 1, no 4, 511-527, 1998.
4.2. Cooke, R.M., E. Jager, D. Lewandowski (2002) Reliability model for underground gas pipelines. *Probabilistic Safety Assessment and Management* E.J. Bonano, A.L. Camp, M.J. Majors, R.A. Thompson (eds), Elsevier, 2002; 1045-1050.
4.3. Cooke, R.M., Eric Jager and D. Lewandowski (2003) Reliability Model for Underground Gas Pipelines. *Case Studies in Reliability and Maintenance*. Edited by Wallace R. Blischke, D.N. Prabhakar Murthy; p. 423-446, ISBN: 0-471-41373-9, 2003, John Wiley and Sons, Inc.
4.4. Cooke, R.M. and Slijkhuis, Karen A. (2003) Expert Judgment in the Uncertainty Analysis of Dike Ring Failure Frequency. *Case Studies in Reliability and Maintenance*. Edited by Wallace R. Blischke, D.N. Prabhakar Murthy; p. 331-352, ISBN: 0-471-41373-9, 2003, John Wiley and Sons, Inc.
4.5. Brown A.J. and Aspinall W.P. (2004) Use of expert opinion elicitation to quantify the internal erosion process in dams. In *Proc: The 13th Biennial British Dams Society Conference*: University of Kent, Canterbury, 22-26th June 2004; 16pp (http://www.britishdams.org/2004conf/synopses/brown.pdf).
4.6. Cooke, R.M. and Goossens, L.H.G.(2004) Expert judgement elicitation for risk assessments of critical infrastructures. *Journal of Risk Research* vol. 7 issue 6, 2004, ISSN 1366-9877, pp 643-657.
4.7. B.J.M. Ale, L.J. Bellamy, A.L.C. Roelen, R.M. Cooke, L.H.J.Goossens A.R. Hale, D. Kurowicka, E. Smith (2005) Development of a causal model for air transport safety. *Proceedings of IMECE2005, 2005 ASME International Mechanical Engineering Congress and Exposition* November 5-11, 2005, Orlando, Florida USA IMECE2005-79374
4.8. Ale, B.J.M. Bellamy, L.J., Boom, R. van der, Cooper, J., Cooke, R.M., Goossens, L.H.J., Hale, A.R., Kurowicka, D., Morales, O. Roelen, A.L.C. and Spouge, J. (2009) Further development of a Causal model for Air Transport Safety (CATS); Building the mathematical heart. *Reliability Engineering & System Safety,* Vol. 94, No. 9. (September 2009), pp. 1433-1441. doi:10.1016/j.ress.2009.02.024 Key: citeulike:5143231
4.9. Ale, B.J.M. (2006), L.J. Bellamy, R.M. Cooke, L.H.J.Goossens, A.R. Hale, A.L.C.Roelen, E. Smith (2006) Towards a causal model for air transport safety – an ongoing research project. *Safety Science* Volume 44, Issue 8, October 2006, Pages 657-673.
4.10. Ale, B., Bellamy, L.J., Cooke, R.M., Duyvis, M., Kurowicka, D. Lin, P.H.,Morales, O., Roelen, A. Spouge, J. (2009) *Causal Model for Air Transport Safety, Final Report*. Ministerie van Verkeer en Waterstaat, Directoraat-Generaal Luchtvaart en Maritieme Zaken, ISBN 10:90 369 1724-7; ISB N 13: 978 90 369 1724-7, The Hague, The Netherlands
4.11. Forys, M.B., Kurowicka, D., Peppelman, B. (2013) A probabilistic model for a gas explosion due to leakages in the grey cast iron gas mains. *Reliability Engineering & System Safety* volume 119, 270 - 279.
4.12. Jaiswal, K.S., Wald, D.J., Perkins, D., Aspinall, W.P. and Kiremidjian, A.S. (2014) Estimating structural collapse fragility of generic building typologies using expert judgment. Chap 117 in: *Safety, Reliability, Risk and Life-Cycle Performance of Structures and Infrastructures* (eds: Deodatis, G., Ellingwood, B.R., Frangopol, D.M.), CRC Press; 879-886.

5. *Information security*

5.1. Ryan, J.C.H. Mazzuchi,T.A. Ryan,D.J., Lopez de la Cruz, J. and Cooke, R.M. (2012) Quantifying information security risks using expert judgment elicitation. *Computers & Operations Research Journal* 39, 774-784. doi:10.1016/j.cor.2010.11.013

6. Natural Hazards

6.1. Aspinall, W.P. (1997) Elicitation of expert opinions for decision-making: concepts, experience and issues. Paper presented to The Royal Society IDNDR conference "Extreme Natural Disasters - Mitigating Strategies for the 21st Century", March 10, 1997; London.
6.2. Aspinall W. and Cooke R.M. (1998) Expert judgement and the Montserrat Volcano eruption. *Proceedings of the 4th International Conference on Probabilistic Safety Assessment and Management PSAM4*, September 13th -18th, 1998, New York City, USA (eds. Ali Mosleh and Robert A. Bari), Vol.3, 2113-2118.
6.3. Aspinall W.P., Loughlin S.C., Michael F.V., Miller A.D., Norton G.E., Rowley K.C., Sparks R.S.J. and Young S.R. (2002) The Montserrat Volcano Observatory: its evolution, organisation, role and activities. In: Druitt, T.H. & Kokelaar, B.P. (eds) *The eruption of Soufrière Hills Volcano, Montserrat, from 1995 to 1999*. Geological Society, London, Memoir.
6.4. Aspinall W.P., Woo G., Voight B. and Baxter P.J. (2003) Evidence-based volcanology: application to eruption crises. *Journal of Volcanology & Geothermal Research*, 123, 273-285.
6.5. Aspinall, W.P. (2006) Structured elicitation of expert judgment for probabilistic hazard and risk assessment in volcanic eruptions. In: Mader, H.M., Coles, S.G., Connor, C.B. & Connor, L.J. (eds) *Statistics in Volcanology*. Special Publications of IAVCEI, 1. Geological Society, London, 15-
6.6. Aspinall, W.P. (2006) Structured elicitation of expert judgment for probabilistic hazard and risk assessment in volcanic eruptions. Chapter 2, in IAVCEI Volume *Statistics in Volcanology*, 31pp.
6.7. Sparks, R.S.J., Aspinall, W.P. et al. (2013) Risk and uncertainty assessment of volcanic hazards. In *Risk and Uncertainty assessment in Natural Hazards*, Rougier, J.C., Sparks R.S.J., Hill, L. (eds). Cambridge University Press, Chapter 11, 364-397.
6.8. Martí, J., W. P. Aspinall, R. Sobradelo, A. Felpeto, A. Geyer, R. Ortiz, P. Baxter, P. Cole, J. Pacheco, M.J. Blanco, C. Lopez (2008) A long-term volcanic hazard event tree for Teide-Pico Viejo stratovolcanoes (Tenerife, Canary Islands). *Journal of Volcanology and Geothermal Research,* 178, 543-552. doi:10.1016/j.jvolgeores.2008.09.023
6.9. Queiroz, G., J. M. Pacheco, J.L. Gaspar, W.P. Aspinall, J.E. Guest and T. Ferreira (2008) The last 5000 years of activity at Sete Cidades volcano (São Miguel Island, Azores): Implications for hazard assessment. *Journal of Volcanology and Geothermal Research,* 178, 562-573. doi:10.1016/j.jvolgeores.2008.03.001
6.10. Neri, A., Aspinall, W.P., Cioni, R., Bertagnini, A., Baxter, P.J., Zuccaro, G., Andronico, D., Barsotti, S., Cole, P.D., Esposti Ongaro, T., Hincks, T.K., Macedonio, G., Papale, P., Rosi, M., Santacroce, R., Woo, G. (2008) Developing an Event Tree for Probabilistic Hazard and Risk Assessment at Vesuvius. *Journal of Volcanology & Geothermal Research,* 178, 397-415. doi:10.1016/j.jvolgeores.2008.05.014
6.11. Aspinall, W.P. (2012) Comment on "Social studies of volcanology: knowledge generation and expert advice on active volcanoes" by Amy Donovan, Clive Oppenheimer and Michael Bravo [*Bull Volcanol* (2012) 74:677-689] *Bulletin of Volcanology*, 74, 1569-1570. doi: 10.1007/s00445-012-0625-x
6.12. Loughlin, S. C., Aspinall, W. P., Vye-Brown, C., Baxter, P. J., Braban, C., Hort, M., Schmidt, A., Thordarson, T., Witham, C. (2012) *Large-magnitude fissure eruptions in Iceland: source characterisation*. BGS Open File Report, OR/12/098; 231pp (available at: http://www.bgs.ac.uk/research/volcanoes/LakiEruptionScenarioPlanning.html)
6.13. Aspinall, W.P., Cooke, R.M. (2013) Expert Elicitation and Judgement. In *Risk and Uncertainty assessment in Natural Hazards*, Rougier, J.C., Sparks R.S.J., Hill, L. (eds). Cambridge University Press, Chapter 4, 64-99.
6.14. Hincks,T.K., Komorowski,J-C., Sparks, S.R., and Aspinall, W.P. (2014) Retrospective analysis of uncertain eruption precursors at La Soufrière volcano, Guadeloupe, 1975-77: volcanic hazard assessment using a Bayesian Belief Network approach *Journal of Applied Volcanology* 2014, 3:3 doi:10.1186/2191-5040-3-3
6.15. Wadge, G. and Aspinall, W.P. (2014) A Review of Volcanic Hazard and Risk Assessments at the Soufrière Hills Volcano, Montserrat from 1997 to 2011. Ch. 24 in: *The Eruption of Soufriere Hills*

Volcano, Montserrat, from 2000 to 2010: Geological Society Memoirs, Vol. 39, (edited by G. Wadge, R.E.A. Robertson and B. Voight). Geological Society: London; 439-456

6.16. Scourse, E., Aspinall, W.P. and Chapman, N. (2014) Using expert elicitation to characterise long-term tectonic risks to radioactive waste repositories in Japan. *Journal of Risk Research*, doi: 10.1080/13669877.2014.971334

7. Climate

7.1. Bamber, J.L., and Aspinall, W.P. (2012) An expert judgement assessment of future sea 1evel rise from the ice sheets. *Nature Climate Change*, published online: January 6, 2012 | DOI: 10.1038/NCLIMATE1778.

8. Banking/Finance

8.1. Van Overbeek, F.N.A. (1999) Financial experts in Uncertainty, Masters Thesis, Department of Mathematics, Delft University of Technology, Delft.

8.2. Qing, X. (2002) Risk analysis for real estate investment, PhD thesis, Dept. of Architecture, TU Delft.

8.3. Bakker. M. (2004) Quantifying Operational Risks within Banks According to Basel II, Masters Thesis, Delft University of Technology, Dept. of Mathematics.

9. Completed, publication in preparation

9.1. Attribution study for Robert Wood Johnson Covering Kids, and Clean Indoor Air in Pennsylvania, conducted by Centers for Disease Dynamics, *Economics & Policy*, 2009.

9.2. Attribution study for Robert Wood Johnson, Covering Kids, Washington, Nebraska, Illinois, Arkansas, Florida, by Centers for Disease Dynamics, *Economics & Policy*, 2012.

9.3. Disease Control Priorities, funded by Bill and Melinda Gates Foundation, Fistula and Obesity, conducted by Centers for Disease Dynamics, *Economics & Policy*, 2013.

9.4. WHO Global Burden of Foodborne Disease (2014)

9.5. Cashman, N.R., Cheung, R., Aspinall, W., Wong, M. and Krewski, D. (2014) Expert Elicitation for the Judgment of Prion Disease Risk Uncertainties associated with Urine-derived and Recombinant Fertility Drugs. Submitted to: *Journal of Toxicology and Environmental Health*.

9.6. Bevilacqua, A., Isaia, R., Neri, A., Vitale, S., Aspinall, W.P. and eight others (2014) Quantifying volcanic hazard at Campi Flegrei caldera (Italy) with uncertainty assessment: I. Vent opening maps. Submitted to *Journal of Geophysical Research - Solid Earth*; AGU.

9.7. Neri, A., Bevilacqua, A., Esposti Ongaro, T., Isaia, R., Aspinall, W.P. and nine others (2014) Quantifying volcanic hazard at Campi Flegrei caldera (Italy) with uncertainty assessment: II. Pyroclastic density current invasion maps. Submitted to *Journal of Geophysical Research - Solid Earth*; AGU.
